# Development of Carbon‐11 Labeled Pyrimidine Derivatives as Novel Positron Emission Tomography (PET) Agents Enabling Brain Sigma‐1 Receptor Imaging

**DOI:** 10.1002/advs.202414827

**Published:** 2025-04-17

**Authors:** Ping Bai, Ashley Gomm, Chi‐Hyeon Yoo, Prasenjit Mondal, Fleur Marie Lobo, Hui Meng, Yanting Zhou, Weiyao Xie, Hsiao‐Ying Wey, Rudolph E. Tanzi, Can Zhang, Changning Wang, Yu Lan

**Affiliations:** ^1^ Department of Respiratory and Critical Care Medicine Targeted Tracer Research and Development Laboratory West China Hospital Sichuan University Chengdu Sichuan 610041 China; ^2^ Institute of Respiratory Health Targeted Tracer Research and Development Laboratory Frontiers Science Center for Disease‐related Molecular Network West China Hospital Sichuan University Chengdu Sichuan 610041 China; ^3^ Precision Medicine Center Precision Medicine Key Laboratory of Sichuan Province West China Hospital Sichuan University Chengdu Sichuan 610041 China; ^4^ The Research Units of West China Chinese Academy of Medical Sciences West China Hospital Chengdu Sichuan 610041 China; ^5^ State Key Laboratory of Respiratory Health and Multimorbidity West China Hospital Chengdu Sichuan 610041 China; ^6^ Genetics and Aging Research Unit McCance Center for Brain Health Mass General Institute for Neurodegenerative Disease Department of Neurology Massachusetts General Hospital Harvard Medical School 114 16th Street Charlestown MA 02129 USA; ^7^ Athinoula A. Martinos Center for Biomedical Imaging Department of Radiology Massachusetts General Hospital Harvard Medical School Charlestown MA 02129 USA; ^8^ Department of Pharmacy Renmin Hospital of Wuhan University Wuhan Hubei 430060 China

**Keywords:** ^11^C‐labeled radioligand, brain PET imaging, pyrimidine derivatives, sigma‐1 receptor (*σ*_1_R)

## Abstract

The sigma‐1 receptor (σ1R) is a stress‐activated chaperone protein that has emerged as a significant therapeutic target for neurodegenerative disorders. Developing effective positron emission tomography (PET) imaging probes targeting σ1R is crucial for visualizing its distribution and function in the brain, as well as facilitating related drug development. In this study, two novel ^11^C‐labeled PET probes based on the structure of a potent σ1R ligand Lan‐0101 are designed and synthesized. PET imaging studies in mice reveal that [^11^C]CNY‐01 exhibits good brain uptake and binding specificity. Subsequent evaluation in non‐human primates further demonstrates that [^11^C]CNY‐01 displays favorable brain penetration, slow clearance kinetics, and characteristics of irreversible binding to its target in blockage experiments. To assess the clinical potential of the probe, both in vitro experiments and in vivo PET imaging using [^11^C]CNY‐01 are conducted in Alzheimer's disease (AD) transgenic mouse models. These studies reveal a significant decrease in σ1R expression in the brain under conditions of AD amyloid pathology and microglial activation, highlighting the probe's sensitivity to disease‐related receptor changes. This work establishes [^11^C]CNY‐01 as a promising tool for investigating the relationship between σ1R and neurological disorders, potentially advancing the understanding of σ1R's role in disease pathophysiology and therapeutic interventions.

## Graphical abstract

Novel carbon‐11 labeled pyrimidine derivatives are developed as PET imaging probes for visualizing sigma‐1 receptors in the brain. [^11^C]CNY‐01 demonstrates superior brain uptake and binding specificity in both rodents and non‐human primates. The study reveals decreased sigma‐1 receptor expression in Alzheimer's disease, offering a promising tool for investigating neurological disorders.

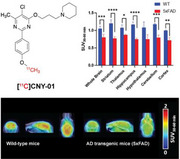

## 1 Introduction

Sigma‐1 receptor (*σ*_1_R) is a chaperon protein mainly expressed intracellular, highly enriched in the endoplasmic reticulum's mitochondrion‐associated membrane (MAM), and modulates several cellular signaling pathways.^[^ ^1^ ^]^ In over 40 years of studies, *σ*_1_R was first incorrectly classified in the G protein‐coupled receptor (GPCR) family of opioid receptors,^[^ ^2^ ^]^ then it was misidentified as the phencyclidine (PCP) receptor, as the deficiency of specific ligands in the early stage of research.^[^ ^3^ ^]^ Further molecular biological and pharmacological studies finally identified that *σ*_1_R is a 223 amino acid‐long protein conserved in vertebrates, but no homology of sequence with any other mammalian proteins.^[^ ^4^ ^]^ Mainly expressed in the central nervous system (CNS),^[^ ^5^, ^6^ ^]^ *σ*_1_R plays a significant role that modulates multiple cellular functions related to neurological disorders, such as amyotrophic lateral sclerosis (ALS),^[^ ^7^, ^8^ ^]^ addiction (e.g., cocaine and alcohol abuse),^[^ ^9^, ^10^ ^]^ Alzheimer`s disease (AD),^[^ ^11^, ^12^, ^13^, ^14^ ^]^ Parkinson's disease (PD),^[^ ^15^, ^16^ ^]^ Huntington's disease (HD),^[^ ^17^ ^]^ depression,^[^ ^18^ ^]^ neuroinflammation, and neuropathic pain.^[^ ^19^, ^20^, ^21^ ^]^

**Figure 1:**
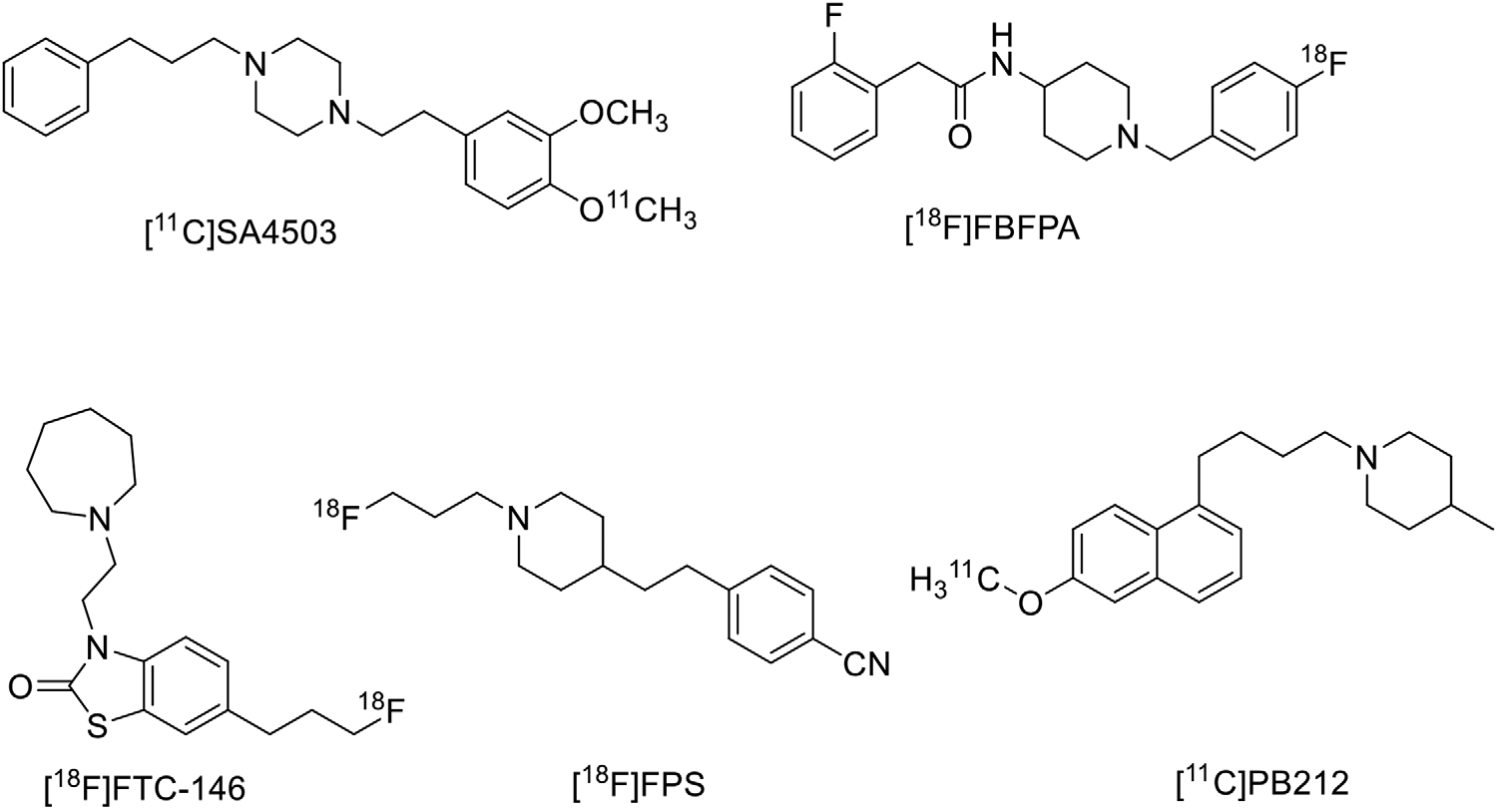
The chemical structures of representative *σ*_1_R PET radiotracers.

The function of *σ*_1_R as a ligand‐operated chaperone protein is regulated by various synthetic ligands with diverse chemical structures despite the fact that no *σ*_1_R endogenous ligands have been fully identified.^[^ ^22^, ^23^ ^]^ Currently, *σ*_1_R ligands have been applied in clinical trials for treating Alzheimer's disease,^[^ ^24^ ^]^ neuropathic pain,^[^ ^25^ ^]^ and ischemic stroke.^[^ ^26^ ^]^ Meanwhile, due to the attractive pharmacological properties, some *σ*_1_R ligands have been translated to radiotracers (**Figure** **1**) for positron emission tomography (PET) imaging, especially for brain PET imaging, which could facilitate the pathophysiology investigating of *σ*_1_R‐related CNS diseases. As the first *σ*_1_R radiotracer applied in clinical research of PET imaging, [^11^C]SA4503 successfully visualized *σ*_1_R in the human brain, despite hurdles and limitations of its clinical application^[^ ^27^ ^]^ caused by the high affinity to *σ*_1_R (K_i_ = 4.0–4.6 nmol L^−1^),^[^ ^28^ ^]^ relatively low selectivity to *σ*_2_R (*σ*_2_R/*σ*_1_R = 13.3–55.0),^[^ ^29^ ^]^ off‐target affinities, i.e., emopamil binding protein (EBP, K_i_ = 1.7 nmol L^−1^),^[^ ^30^ ^]^ and vesicular acetylcholine transporter (VAChT, K_i_ = 50 nmol L^−1^). The fluorine‐18‐labeled *σ*_1_R radioligands have also been developed, such as ^18^F‐fluoromethyl analog of the SA4503 ([^18^F]FMSA4503)^[^ ^31^ ^]^ and [^18^F]FTC‐146.^[^ ^32^ ^]^ [^18^F]FTC‐146 has recently completed an early phase I trial of PET/MRI, and in complex regional pain syndrome (CRPS) and sciatica.^[^ ^33^ ^]^ [^18^F]FTC‐146 has good binding selectivity and high uptake in the human brain during a three‐hour imaging period. Still, unexpectedly slow brain clearance was observed, possibly due to the picomolar range *σ*_1_R binding affinity (K_i_ = 0.0025 nmol L^−1^).^[^ ^34^ ^]^ Despite the relentless efforts in *σ*_1_R PET radiotracer development, no radiotracer in progress has met the clinical requirements of practical application in human brain imaging and diagnosis so far.

To develop *σ*_1_R PET imaging radiotracers for clinical practice, our research started with the design and synthesis of potent and selective *σ*_1_R ligands. In our previous work, a series of *σ*_1_R selective ligands with the pyrimidine scaffold was identified through the virtual screen using a three‐dimensional‐quantitative structure‐active relationship (3D‐QSAR) pharmacophore model.^[^ ^35^ ^]^ The most promising compound, 5‐chloro‐2‐(4‐chlorophenyl)‐4‐methyl‐6‐(3‐(piperidin‐1‐yl)propoxy)pyrimidine (Lan‐0101), exhibited a specific binding affinity to *σ*_1_R (*σ*_1_R K_i_ = 1.06 nmol L^−1^, *σ*_2_R/*σ*_1_R = 1344), and no significant binding affinities to non‐target receptors or ion channels. Moreover, as a *σ*_1_R antagonist, Lan‐0101 showed good brain penetration and exerted dose‐dependent antinociceptive effects in rodent acute and neuropathic pain models.^[^ ^35^ ^]^ These results indicate that Lan‐0101 has the potential to be developed as a radioligand for brain *σ*_1_R imaging. In this work, we report two novel *σ*_1_R PET radioligands, [^11^C]CNY‐01 and [^11^C]CNY‐02, based on Lan‐0101. PET imaging studies of [^11^C]CNY‐01 and [^11^C]CNY‐02 in rodents showed that both radiotracers had good brain uptake, while [^11^C]CNY‐01 exhibited superior specific binding. We further evaluated the in vivo performance of [^11^C]CNY‐01 in a non‐human primate (NHP), which showed the safety profile and translational potential of [^11^C]CNY‐01. More importantly, we carried out PET imaging studies of [^11^C]CNY‐01 in AD transgenic mice model, and the results demonstrated the potential of [^11^C]CNY‐01 in the diagnosis and investigation of the related pathological mechanisms of AD. This work provided a new tool for studying the relationship between *σ*_1_R and neurological diseases and laid the foundation for further development of *σ*_1_R radiotracers with clinical application potential.

## 2 Result and Discussion

### 2.1 PET Probes Design and Standard Compound Synthesis

**Figure 2:**
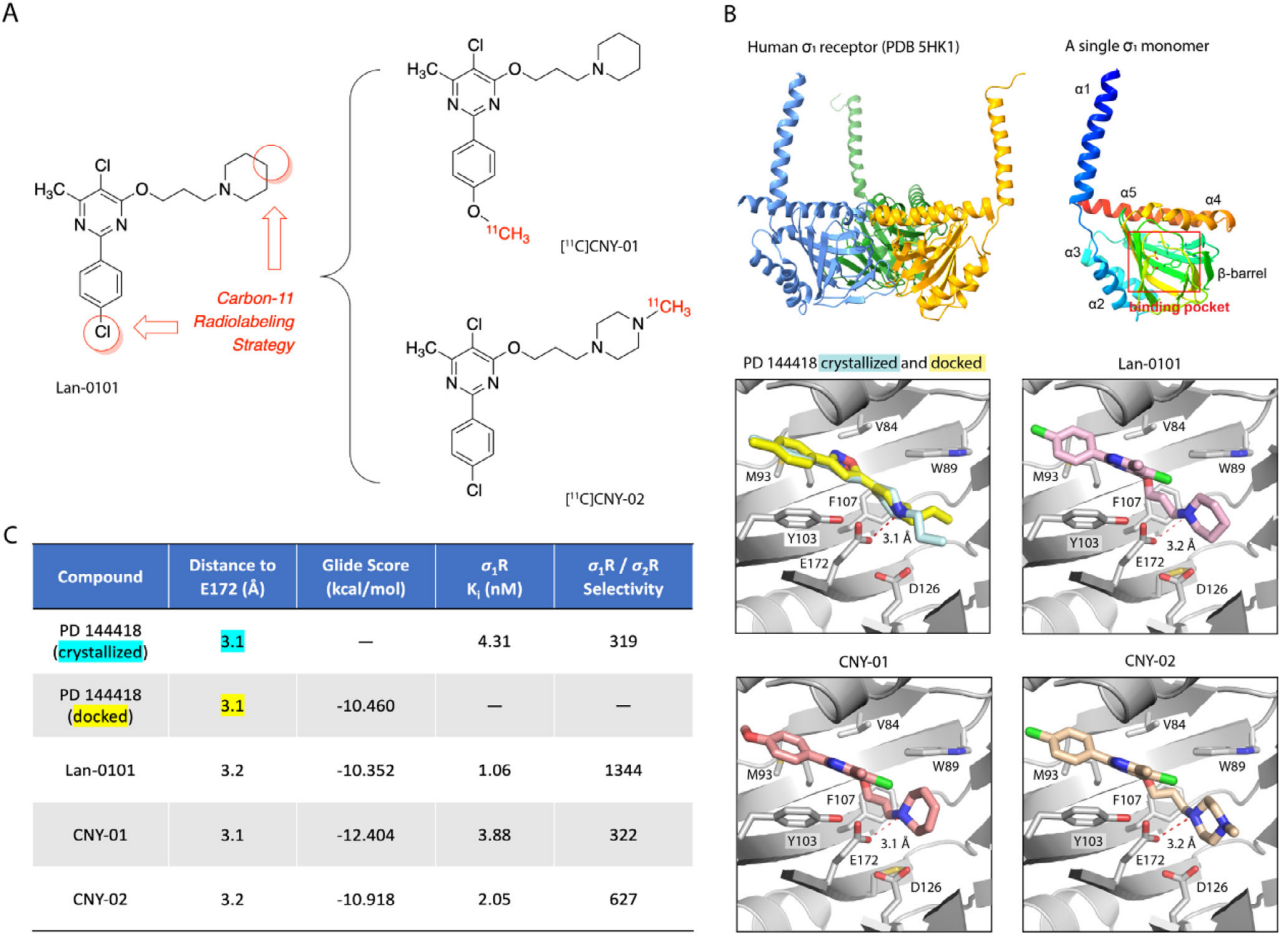
The chemical design and radiolabeling strategy of [^11^C]CNY‐01 and [^11^C]CNY‐02. A) Carbon‐11 radiolabeling strategy based on Lan‐0101, to acquire two new probes [^11^C]CNY‐01 and [^11^C]CNY‐02. B) The overall structure of human *σ*_1_R (PDB: 5HK1), the structure of a single *σ*_1_ monomer, and Glide docking of compounds into the *σ*_1_R binding pocket for analysis. Pose of co‐crystallized PD 144 418 (pale cyan) and top‐ranked pose (yellow). Best docked poses for Lan‐0101 (lavender), CNY‐01 (salmon), and CNY‐02 (light gold). In all panels, the receptor is shown in gray. C) Glide docking results and ex vivo binding affinities for PD 144 418, Lan‐0101, CNY‐01 and CNY‐02.

Due to the lack of suitable isotope labeling sites in the structure, minor chemical modifications were made to Lan‐0101 (**Figure** **2**A). We first utilized molecular docking to investigate the interaction mode between Lan‐0101 and the *σ*_1_R (PDB code: 5HK1).^[^ ^36^ ^]^ As shown in Figure 2B, Lan‐0101 overlaps significantly with the native ligand PD 144418^[^ ^37^ ^]^ (a known selective *σ*1R ligand) and exhibits similar interactions with the protein. Further analysis revealed that the pyrimidine core of Lan‐0101 is a crucial group interacting with the protein's binding pocket (Figure 2B). The nitrogen atom of the piperidine ring can form hydrogen bonds with the hydrophobic region of the protein's E172 residue. Based on this, we employed the bioisostere replacement strategy to design compounds CNY‐01 and CNY‐02 analogs, aiming to maintain the compounds' binding affinity towards *σ*_1_R while facilitating carbon‐11 labeling (Figure 2A). Additionally, molecular simulations were conducted for CNY‐01 and CNY‐02, revealing that their ligand‐protein interactions closely resemble those of Lan‐0101 and with good Glide Scores (Figure 2C), which implies their equipotency in bioactivity.

**Scheme 1:**
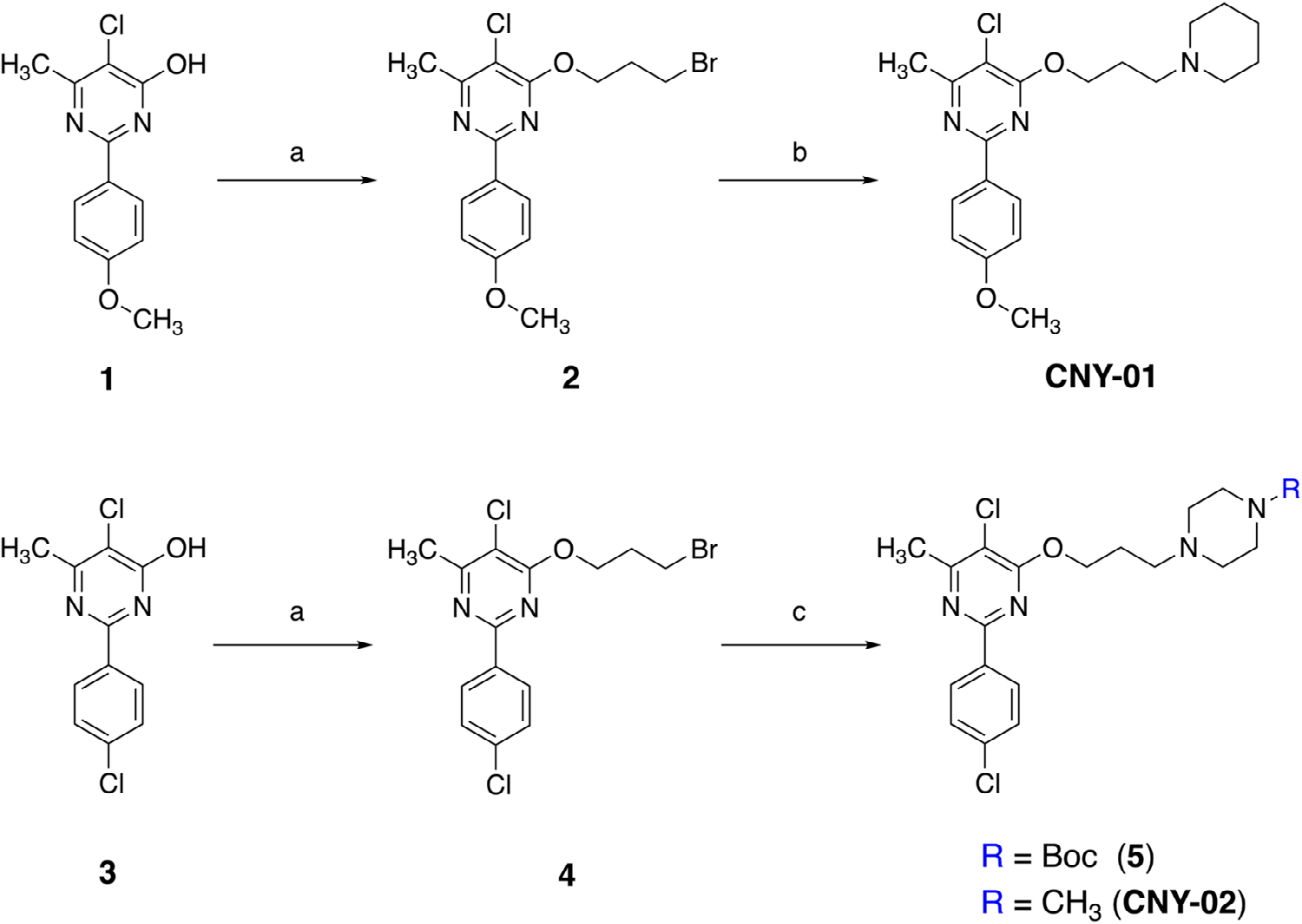
Synthesis of standard CNY‐01 and CNY‐02. a) 1,3‐dibromopropane, potassium carbonate, acetone, 58 °C, 2.0 h; b) piperidine, cesium carbonate, acetonitrile, 82 °C, 4.0 h; c) 1‐methylpiperazine, cesium carbonate, acetonitrile, 82 °C, 4.0 h.

Following the method reported previously,^[^ ^35^ ^]^ using the 5‐chloro‐2‐(4‐methoxyphenyl)‐6‐methylpyrimidin‐4‐ol analogs as the starting materials, the CNY‐01 and CNY‐02 were prepared after a two‐step nucleophilic substitution reaction (**Scheme** **1**).

### 2.2 *σ*Rs Binding Studies of CNY‐01 and CNY‐02

The binding potential (BP) is a significant character that is calculated as the receptor's density (evaluated in *B*_max_) relative to the specific ligand's binding affinity. Radiotracers with a BP value greater than 5 are often preferred because they demonstrate higher target‐specific binding affinity and better signal‐to‐noise ratio in imaging applications.^[^ ^38^ ^]^ The *B*_max_ of *σ*_1_R in the human brain was estimated to be 3‒60 nmol L^−1^ (30‒600 fmol mg^−1^).^[^ ^39^, ^40^ ^]^ The anatomical distribution of *σ*_1_R in the brain was also analyzed by using [^3^H]haloperidol or [^3^H]pentazocine. The highest concentrations of these sigma binding sites were in the cerebellar cortex, accumbens nucleus, and cortical regions.^[^ ^39^, ^40^ ^]^ Besides, the region's distribution of *σ*_1_R in mouse brains was also determined by multiple methods, such as using immunohistochemistry with a specific antibody. High levels of *σ*_1_R immunostaining were associated with neurons in specific brain regions, including the olfactory bulb, several hypothalamic nuclei, the septum, the central gray, and certain motor nuclei of the hindbrain.^[^ ^41^, ^42^ ^]^ Based on the *B*_max_ of *σ*_1_R, the suggested suitable binding affinity range for radiotracers applied in the brain *σ*_1_R imaging is 0.3‒6 nmol L^−1^.

The ex vivo binding affinities of *σ*Rs of CNY‐01 and CNY‐02 were measured using a competitive radioligand binding assay.^[^ ^35^ ^]^ As a result, both CNY‐01 (*σ*_1_R K_i_ = 3.88 nmol L^−1^, *σ*_2_R/*σ*_1_R = 322) and CNY‐02 (*σ*_1_R K_i_ = 2.05 nmol L^−1^, *σ*_2_R/*σ*_1_R = 627) showed high binding affinities and selectivity to *σ*_1_R, satisfying the criteria of suitable affinity for brain *σ*_1_R imaging. Besides, both CNY‐01 and CNY‐02 showed good binding selectivity, with no significant bindings to 43 CNS‐related targets at the concentration of 10 *µ* m (Psychoactive Drug Screening Program (PDSP) screening, Table S1, Supporting Information).

### 2.3 Radiosynthesis

**Scheme 2:**
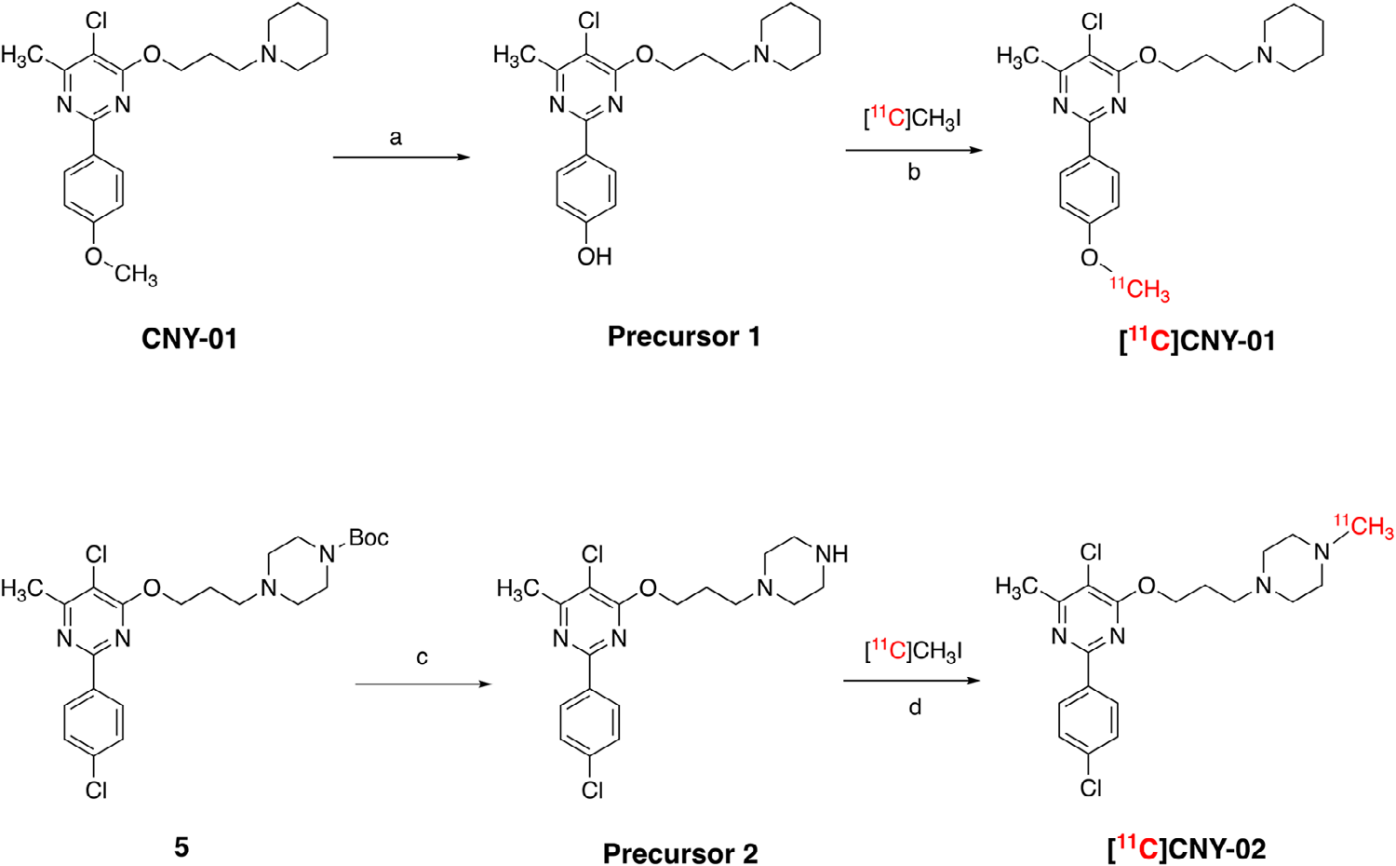
Precursor preparation and radiosynthesis of [^11^C]CNY‐01 and [^11^C]CNY‐02. a) BBr_3_, dichloromethane, ‒78 °C to room temperature, overnight; b) NaOH, dimethyl formamide, 120 °C, 3.0 min; c) TFA, dichloromethane, room temperature, 2.0 h; d) K_2_CO_3_, dimethyl formamide, 120 °C, 3.0 min.

[^11^C]CNY‐01 and [^11^C]CNY‐01 were radiosynthesized through a methylation reaction using the corresponding precursor (**Scheme** **2**).^[^ ^43^ ^]^ The precursor was reacted with [^11^C]CH_3_I in the presence of base at 120 °C for 3.0 min to obtain the crude radiotracer. Following the separation and purification in semipreparative HPLC, [^11^C]CNY‐01 and [^11^C]CNY‐02 were obtained with moderate radiochemical yields (6%‒15%, non‐decay corrected) at the end of synthesis (EOS) and high specific activity of 59.2 ± 7.4 GBq/*µm*ol and 45.1 ± 3.2 GBq/*µm*ol (EOB), respectively.

### 2.4 Preliminary Mice PET Imaging

**Figure 3:**
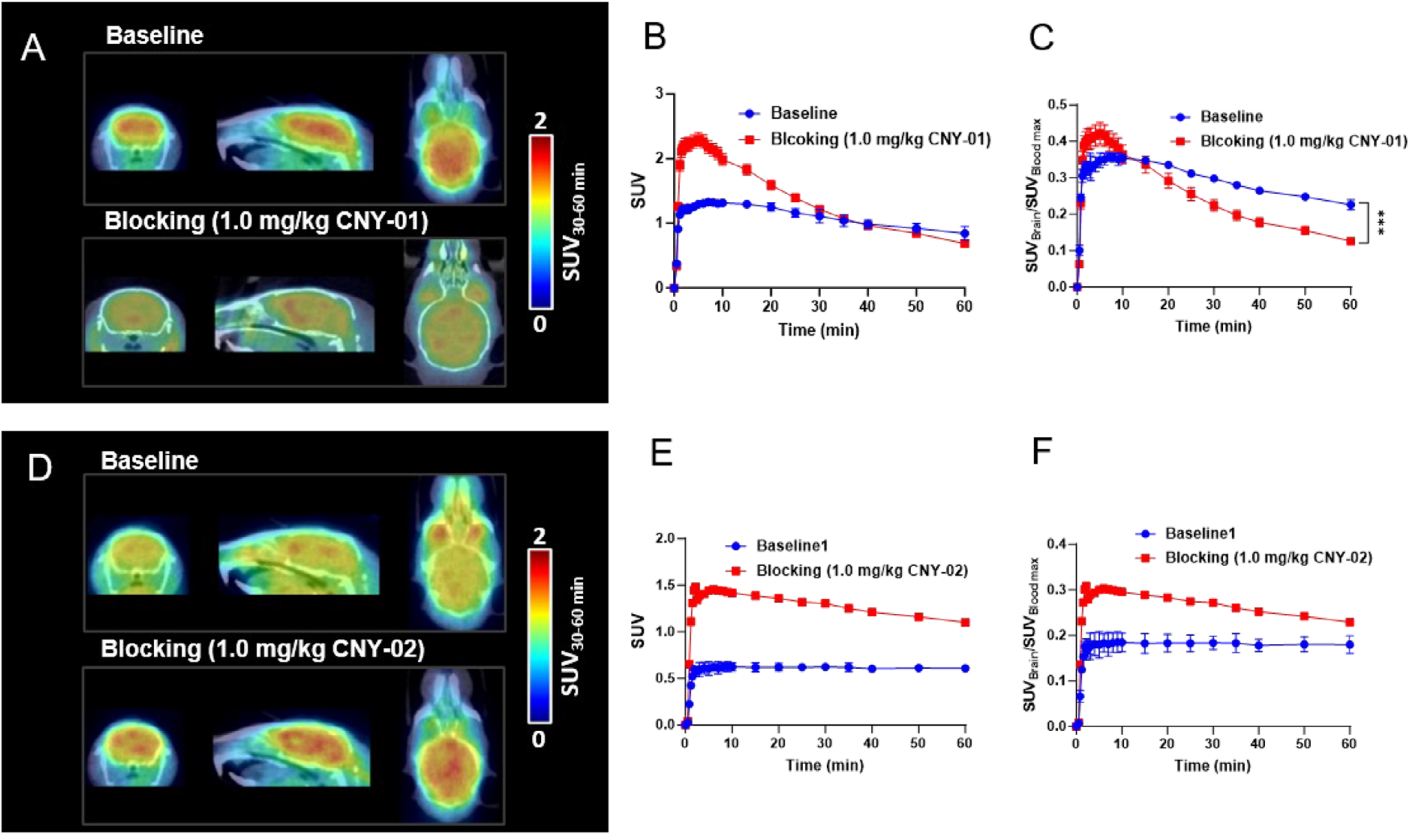
Mice PET/CT study with [^11^C]CNY‐01 and [^11^C]CNY‐02. A) Representative baseline and blocking (pre‐treated with 1.0 mg kg^−1^ CNY‐01) PET/CT images of [^11^C]CNY‐01 focused on the brain (summed 30‒60 min, n = 4). B) Whole brain time‐activity curves of [^11^C]CNY‐01 for baseline and blocking mice. C) The normalized baseline and blocking TACs of [^11^C]CNY‐01 (normalized the brain uptake curves with the maximum radioactivity in the blood at each time point). D) Representative baseline and blocking (pre‐treated with 1.0 mg kg^−1^ CNY‐02) PET/CT images of [^11^C]CNY‐02 focused on the brain (summed 30‒60 min, n = 4). E) Whole brain time‐activity curves of [^11^C]CNY‐02 for baseline and blocking mice. F) The normalized baseline and blocking TACs of [^11^C]CNY‐02 (normalized the brain uptake curves with the maximum radioactivity in the blood at each time point). Data were presented as mean ± standard error of the mean (SEM). Quantification of results was performed using Student's *t*‐test (***p < 0.0001).

PET/CT imaging studies of [^11^C]CNY‐01 and [^11^C]CNY‐02 were performed in male C57BL/6 mice. As shown in **Figure** **3**, both [^11^C]CNY‐01 and [^11^C]CNY‐02 could penetrate the BBB, with the maximum standard uptake values (SUVs) of 1.3 and 0.7 in the brain, respectively (Figure 3). The whole‐brain time‐activity curve (TAC) of each tracer was generated. Of note, [^11^C]CNY‐01 exhibited higher brain uptake and better brain clearance kinetics than [^11^C]CNY‐02 within the scan time frame. To investigate the binding specificity of [^11^C]CNY‐01 and [^11^C]CNY‐02, blocking studies were carried out by pre‐administrating mice with corresponding unlabeled ligands. As a result, The TAC indicates that pre‐injection of unlabeled CNY‐01 resulted in an initial increase in maximum radioactivity uptake in the mouse brain compared to the baseline, which then rapidly decreased over time (Figure 3B). This is due to the receptors in peripheral tissues being occupied by CNY‐01, leading to more radiotracer entering the brain and quickly washing out. However, pre‐treatment of CNY‐02 resulted in a significantly increased brain uptake of [^11^C]CNY‐02 without blocking effects (Figure 3E), which indicates the non‐specificity binding of [^11^C]CNY‐02 in vivo. Of note, the TACs show an increase in brain tracer uptake during the blocking studies, which could be attributed to peripheral blocking effects. To address this, we normalized the brain uptake data to the maximum radioactivity observed in the blood (input function). This normalization allowed us to account for peripheral changes in tracer availability. As shown in Figure 3C,F, the normalized data demonstrate that [^11^C]CNY‐01 exhibits a significant blocking effect, confirming its good binding specificity. In contrast, [^11^C]CNY‐02 still does not show a significant blocking effect even after normalization. These results further validate the binding specificity of [^11^C]CNY‐01.

### 2.5 PET Imaging of [^11^C]CNY‐01 in NHP

**Figure 4:**
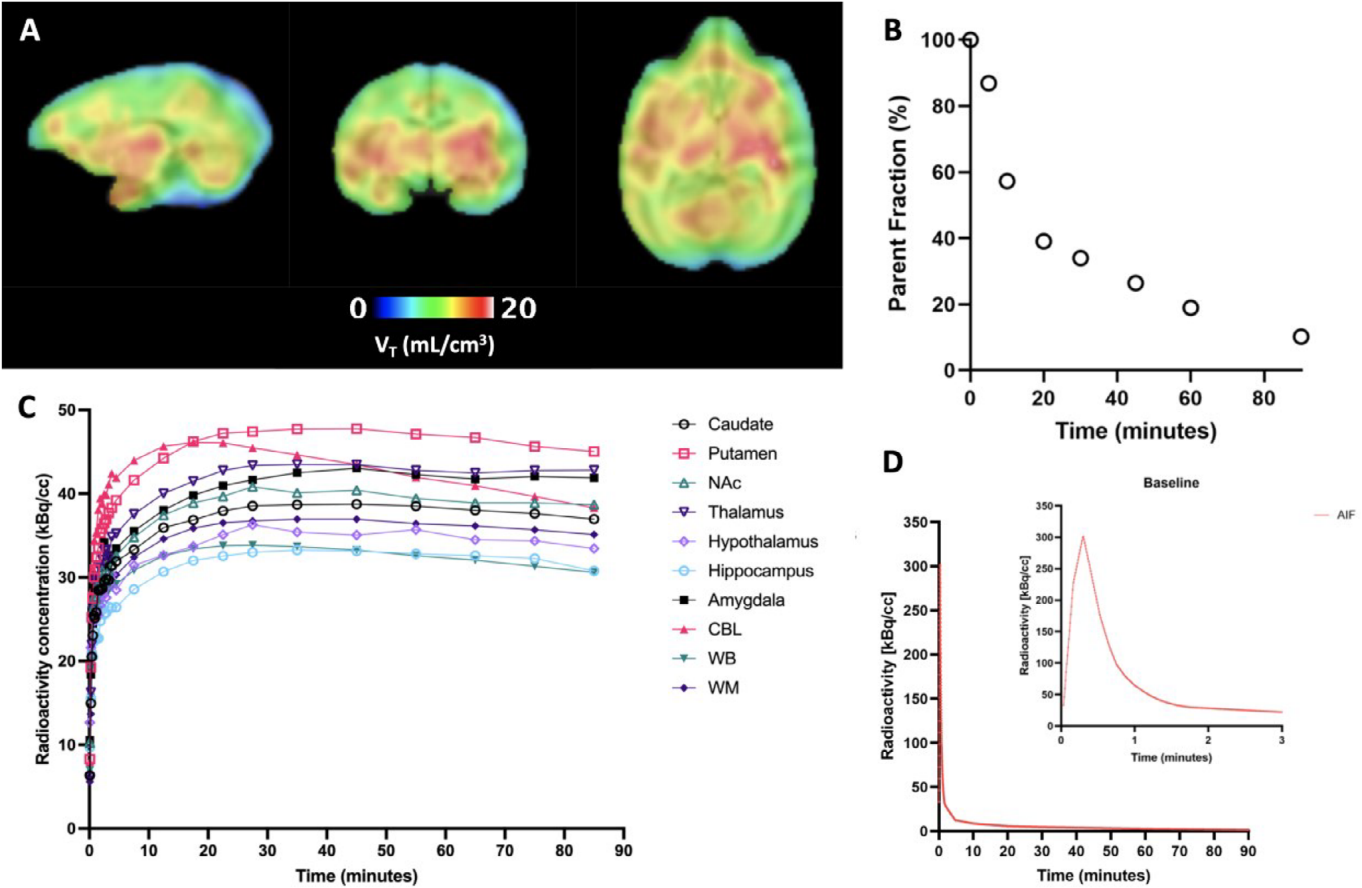
PET/MR imaging studies of [^11^C]CNY‐01 in NHP. A) The parametric volume of interest (V_T_) map of [^11^C]CNY‐01 focused on the monkey brain, estimated by using the Logan graphical method with metabolite‐corrected arterial input function illustrated with B) parent fraction of [^11^C]CNY‐01. C) Time‐activity curves of [^11^C]CNY‐01 in brain regions of interest, illustrated with D) radio metabolite‐corrected plasm radioactivity.

Next, we conducted PET/MR imaging studies using [^11^C]CNY‐01 in non‐human primates (NHP) to evaluate its safety and translational potential in large animals further. A 90‐minute dynamic PET scan with [^11^C]CNY‐01 focusing on the head of a male rhesus monkey. We performed a full kinetic analysis with one‐ and two‐tissue compartment models (1TCM and 2TCM), as well as logan graphical analysis (LGA), using metabolite‐corrected arterial input function (AIF), similar to previous studies,^[^ ^44^, ^45^ ^]^ to determine which model best suit kinetics of [^11^C]CNY‐01. In most brain regions, 2TCM provided a better fit than 1TCM, as indicated by the Akaike information criterion (AIC) and model selection criterion (MSC) (Table 1). **Figure** **4**A shows that [^11^C]CNY‐01 exhibited favorable brain uptake, with a volume of distribution (V_T_) of 10.35 mL cm^−^^3^ within the whole brain, derived from 2TCM. Figure 4C displays TACs for key brain regions of interest, including the caudate, putamen, nucleus accumbens (NAc), thalamus, hypothalamus, midbrain, amygdala, hippocampus, cerebellum (CBL), white matter (WM), and whole brain (WB). The highest V_T_, 19.46 mL cm^−^^3^, was observed in the thalamus, followed by the anterior cingulate cortex (ACC; 15.87 mL cm^−^^3^), putamen (15.75 mL cm^−^^3^), white matter (15.00 mL cm^−^^3^), globus pallidus (14.90 mL cm^−^^3^), and midbrain (14.71 mL cm^−^^3^) in descending order. To test whether non‐parametric graphical approaches can be used for [^11^C]CNY‐01, we performed LGA with an assumption of steady‐state time (T*) of 40 mins (Table 1). We found that regional V_T_ derived from 2TCM and LGA were in close agreement across the brain (Pearson *r* = 0.8860; *p* < 0.0001). For voxel‐wise estimation of V_T_ across the whole brain, we then used LGA (Figure 4A). Notably, relatively higher [^11^C]CNY‐01 uptake was observed in the putamen and midbrain, aligning with the high expression of *σ*_1_ receptors (*σ*_1_R) in these regions (Figure 4C). Conversely, regions like the NAc, which exhibit low *σ*_1_R expression, showed lower [^11^C]CNY‐01 uptake.

**Table 1:** Regional volume of distribution (V_T_) values obtained from a male NHP brain.

Region	One tissue compartment model [1TCM]			Two tissue compartment model [2TCM]			Logan graphical method [T* = 40 min]
	V_T_ [mL cm^−3^]	AIC	MSC	V_T_ [mL cm^−3^]	AIC	MSC	V_T_ [mL cm^−3^]
Caudate	9.58	21.84	2.47	14.24	−0.92	3.40	12.71
Putamen	11.56	14.92	2.73	15.75	−34.90	4.70	15.02
Globus‐pallidus	11.32	21.83	2.40	14.90	−30.43	4.46	15.11
NAc	9.85	22.82	2.22	11.85	3.05	3.03	13.11
VTA	8.25	31.33	1.58	11.16	10.68	2.43	12.11
Thalamus	10.94	23.49	2.48	19.46	−3.98	3.59	15.50
Hypothalamus	8.36	33.71	1.40	10.60	15.59	2.15	11.42
ACC	9.76	23.21	2.49	15.87	6.42	3.19	13.32
PCC	9.29	18.97	2.60	14.68	−5.58	3.60	12.38
Insula	11.17	24.41	2.17	13.96	−27.62	4.22	14.70
Midbrain	11.03	16.02	2.75	14.71	−9.80	3.80	13.39
Sensory	6.73	30.82	1.86	9.33	16.08	2.48	8.75
Motor	6.97	33.54	1.44	8.88	−19.43	3.53	9.39
Ifc	7.36	28.43	1.99	10.89	8.10	2.82	9.62
Ofc	7.31	32.17	1.46	9.24	−20.76	3.55	9.72
Occg	5.24	46.62	0.51	6.28	−9.75	2.73	7.07
Hippocampus	8.04	20.83	2.17	10.36	1.28	2.97	10.31
Amygdala	10.83	29.56	2.00	13.78	1.36	3.14	14.75
CBL	8.96	28.97	1.88	11.68	−26.70	4.08	11.65
WB	7.44	24.65	2.03	10.35	−7.14	3.30	9.82
WM	9.00	25.31	2.35	15.00	8.94	3.03	11.98

Understanding the metabolism of [^11^C]CNY‐01 is crucial for modeling its distribution kinetics and evaluating safety profiles. Figure 4B illustrates the presence of radiometabolites in arterial plasma following a bolus injection of [^11^C]CNY‐01. Ten minutes after administration, less than 10% of the total radioactivity in arterial plasma was attributed to [^11^C]CNY‐01 (Figure 4D), suggesting rapid clearance of the radiotracer from the bloodstream. We also observed that HPLC analysis revealed ≈90% of [^11^C]CNY‐01′s metabolites in plasma are highly polar metabolites (Figure S5, Supporting Information), which are unlikely to cross the BBB, and cause nonspecific binding. This characteristic makes it well‐suited for kinetic modeling and reducing background signal interference. Furthermore, an assessment of [^11^C]CNY‐01 stability in plasma revealed the sustained presence of over 30% of the parent compound at the 30‐minute mark (Figure 4D).

We also performed a self‐blocking scan for [^11^C]CNY‐01, administering 0.5 mg kg^−1^ of unlabeled CNY‐01 10 min before the radiotracer. The metabolism of the radiotracer in the blocking scan was similar to that of the baseline (Figure S6, Supporting Information). The radioactivity concentration was lower in arterial blood samples in the blocking scan compared to the baseline, while brain uptake was higher in the blocking scan (Figure S6, Supporting Information). These differences led to higher V_T_ across the brain in the blocking scan compared to the baseline (Table S2, Supporting Information). The variation of the radiotracer plasma protein binding (fp) between baseline and blocking scan may explain our observation in a way similar to that of the previous study.^[^ ^46^ ^]^ Nonetheless, due to this study's lack of fp data, the exact explanation for our observation remains to be determined further.

The previously reported irreversible binding *σ*_1_R radiotracers, such as [^11^C]SA5845, exhibit distinct metabolic and pharmacokinetic characteristics due to their covalent or near‐covalent interaction with target proteins.^[^ ^47^, ^48^ ^]^ Irreversible tracers typically exhibit slower clearance and relatively high nonspecific binding compared to reversible tracers. In our monkey experiments, [^11^C]CNY‐01 demonstrated a pattern characteristic of irreversible tracers, with gradual brain uptake reaching a plateau followed by slow washout, as well as high nonspecific binding observed in blockage experiments. Based on these pharmacokinetic characteristics, we speculate that within the timeframe of our imaging studies, the binding of [^11^C]CNY‐01 to its target is predominantly irreversible. While we plan to further investigate its binding kinetics in future studies, at the current stage, these irreversible‐like properties should be carefully considered when interpreting quantitative parameters derived from the imaging data and when designing future applications of this radioligand.

### 2.6 PET Imaging of [^11^C]CNY‐01 in AD Transgenic Model

**Figure 5:**
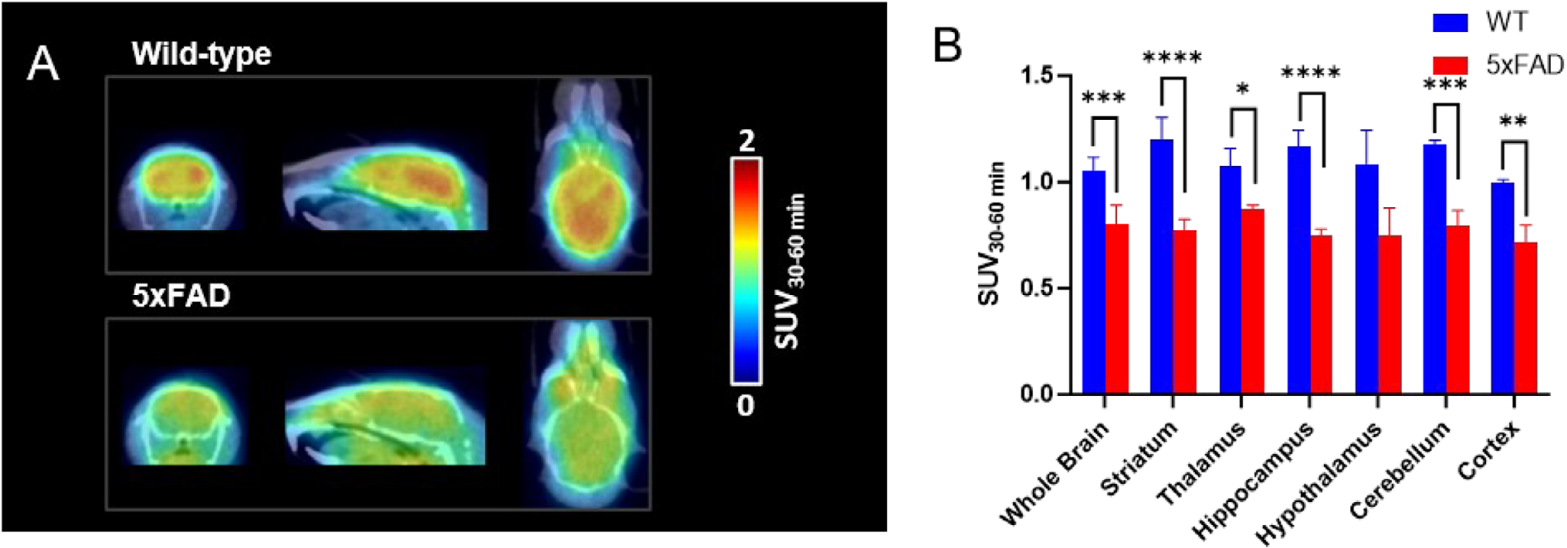
PET/CT imaging studies of [^11^C]CNY‐01 in WT and 5xFAD mouse model. A) Representative PET/CT images (30–60 min) focused on the WT and 5xFAD mice brain. (n = 4). B) The brain uptake of [^11^C]CNY‐01 shows significantly decreased *σ*1R expression in the brain of 5xFAD mice. Data were presented as mean ± standard error of the mean (SEM). Quantification of results was performed using Student's *t*‐test (****p < 0.0001, ***p < 0.001, **p < 0.01, **p < 0.05).

Numerous studies have shown that *σ*_1_R is abnormally expressed in the AD brain, and targeting *σ*_1_R becomes a promising strategy for diagnosis and treatment of AD.^[^ ^11^, ^49^ ^]^ To evaluate the diagnostic potential of [^11^C]CNY‐01 for AD and its potential application in studying the relationship between the *σ*_1_R and AD pathology, we conducted PET imaging studies on 5xFAD transgenic mice model and the corresponding wild‐type (WT) mice. The studies showed a significantly lower uptake of [^11^C]CNY‐01 in the brains of 5xFAD mice compared to WT mice (**Figure** **5**A). Of particular note, brain regions with relatively high *σ*_1_R expression observed higher reduction of radioactivity uptake, such as the striatum, hippocampus, and cerebellum, indicating a decrease in *σ*_1_R expression in the brain under AD pathological conditions, consistent with previous reports (Figure 5B).^[^ ^11^, ^49^, ^50^ ^]^ This result demonstrates that our radioligand can provide a tool for the diagnosis and research of AD and sigma1‐related neurological diseases.

### 2.7 Ex vivo Studies of *σ*_1_R Expression Changes in AD Transgenic Animals

**Figure 6:**
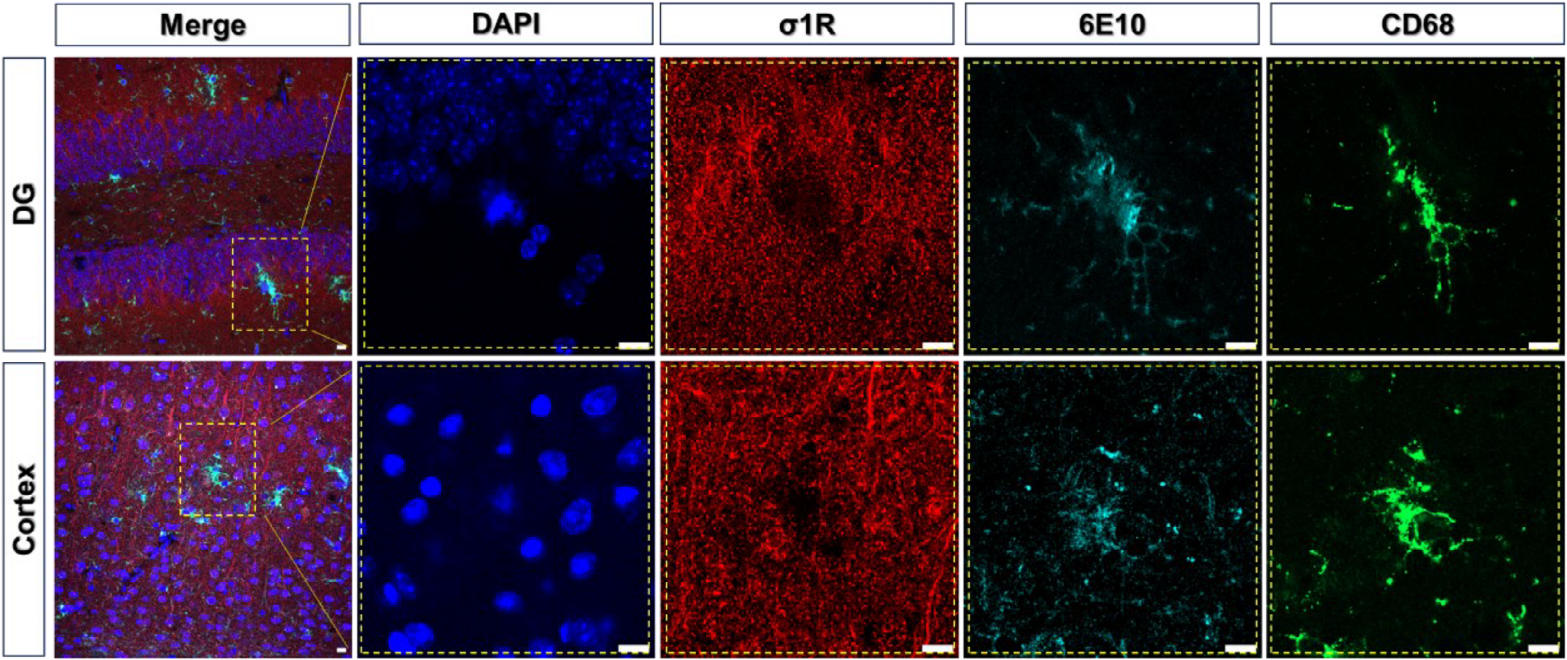
Fluorescent immunohistochemistry analysis of *σ*_1_R in association with amyloid pathology in 5xFAD transgenic animal brain sections. The analysis was performed on 5‐month‐old male 5xFAD mice brain sections stained with *σ*_1_R, in combination with DAPI (for nuclei), 6E10 (for amyloid deposition), and CD68 (microglia activation).

Subsequently, we conducted immunohistochemistry (IHC) assessments specifically targeting *σ*_1_R changes related to AD (**Figure** **6**; Figure S1, Supporting Information). Although AD has been well‐characterized with amyloid pathology as well as microglia activation and neuroinflammation, the expression of *σ*_1_R as an endoplasmic reticulum (ER) stress protein in AD brains remains to be further analyzed. We performed IHC on both WT and 5xFAD mouse brain sections using *σ*_1_R as the primary antibody co‐stained with the antibodies respectively responsive to amyloid plaques and microglia activation. Our results demonstrated *σ*_1_R expression in various AD‐related brain regions, including the hippocampal dentate gyrus (DG) and cortex (Figure 6). Interestingly, we observed a reduction in *σ*_1_R signal in the presence of amyloid deposition in 5xFAD mice. Additionally, microglia activation and distorted nuclei were associated with amyloid deposition in these mice (Figure 6). Furthermore, we performed IHC analysis of *σ*1R in WT non‐transgenic and 5xFAD transgenic animal brain sections, which were captured by C2 microscope z‐stack images co‐stained for *σ*_1_R, in combination with amyloid deposition, microglia activation, and for nuclei. We assessed both WT and AD brain sections and generated z‐stack images of 40× magnifications in both orthogonal projections (Figure S1, Supporting Information) and frame‐by‐frame montages (Figure S2, Supporting Information). In contrast to the hallmark amyloid depositions in the brains of AD mice, we did not find amyloid deposition in wild‐type animals as expected (Figures S1 and S2, Supporting Information). Additionally, we showed both amyloid deposition‐driven microglia activation and the absence of *σ*_1_R by amyloid deposition in AD brains, but not WT brains (Figures S1 and S2, Supporting Information). Moreover, we performed further in‐depth z‐stack imaging analysis of both WT and AD mouse brain sections and generated XY plane composite Movie S1 (Supporting Information) (Figure S3, Supporting Information) and 360 rotation Movie S2 (Supporting Information) (Figure S4, Supporting Information), which displayed dynamic localizations of *σ*_1_R in combination with amyloid deposition and microglia activation in AD. Collectively, our ex vivo IHC data aligned with our PET imaging findings, suggesting that amyloid deposition‐associated *σ*_1_R represents an important neuropathological phenotype in AD.

## 3 Conclusion

In summary, two ^11^C‐radioligands of pyrimidine derivatives targeting *σ*_1_R were successfully developed through a straight and reliable procedure of ^11^C‐labeling, and then evaluated in vivo in rodents. Both radioligands can penetrate BBB and bind to *σ*_1_R in mice brains. Of note, [^11^C]CNY‐01 exhibited more favorable binding specificity and kinetic properties. The differential uptakes of [^11^C]CNY‐01 in the major functional regions of the brain, matched the expression and distribution of *σ*_1_R as reported previously, demonstrating its selective binding towards *σ*_1_R. Subsequent studies conducted in NHP have provided further insights into the safety and potential clinical applications of [^11^C]CNY‐01. Furthermore, we carried out PET imaging of [^11^C]CNY‐01 in transgenic mouse models of AD, and an array of in vitro experiments found a notable decrease in *σ*_1_R expression within the brain in the context of AD pathology. By shedding light on the molecular changes associated with AD pathology, this research paves the way for potential advancements in diagnostic and therapeutic strategies targeting *σ*1R in neurological disorders.

## 4 Experimental Section

### General Procedures

All commercially available chemical reagents and solvents used in the experiments were of ACS‐grade purity or higher and were utilized without further purification. Anhydrous dimethyl sulfoxide and acetonitrile were sourced from Acros Organics. Starting compounds 5‐chloro‐2‐(4‐methoxyphenyl)‐6‐methylpyrimidin‐4‐ol (compound **1**) and 5‐chloro‐2‐(4‐chlorophenyl)‐6‐methylpyrimidin‐4‐ol (compound **4**) were synthesized previously and stored at −20 °C before use. NMR spectra data were recorded on a JEOL JNM‐ECZ500R Spectrometer operating at 500 MHz for ^1^H and 126 MHz for ^13^C. Chemical shifts are reported in δ values (ppm), with tetramethylsilane (TMS) serving as the internal standard. Coupling constants (*J*) are provided in Hz, with signal multiplicities characterized as follows: s (singlet), d (doublet), t (triplet), q (quartet), m (multiplet), and br (broad signal). Analytical thin layer chromatography (TLC) was conducted using silica gel GF254. Column chromatography purification was carried out using silica gel as the stationary phase. Analytical separations were performed on an Agilent 1100 series HPLC system equipped with a diode‐array detector, quaternary pump, vacuum degasser, and autosampler. Mass spectrometry data were obtained using an Agilent 6310 ion trap mass spectrometer (equipped with an ESI source) connected to an Agilent 1200 series HPLC system featuring a quaternary pump, vacuum degasser, diode‐array detector, and autosampler.

[^11^C]CO_2_ (1.2 Ci) was produced via the ^14^N (*p, α*) ^11^C reaction on nitrogen with 2.5% oxygen using 11 MeV protons on a Siemens Eclipse cyclotron (Siemens Healthcare GmbH, Erlangen, Germany). The [^11^C]CO_2_ was trapped on molecular sieves in a TRACERlab FX‐MeI synthesizer (General Electric, GE Healthcare, Boston, MA, USA). [^11^C]CH_4_ was synthesized by reducing [^11^C]CO_2_ in the presence of nickel and hydrogen at 350 °C. The [^11^C]CO_2_ was recirculated through an oven containing iodine (I_2_) to produce [^11^C]CH_3_I via a radical reaction.

All animal studies were conducted at Massachusetts General Hospital (PHS Assurance of Compliance No. A3596‐01). The Subcommittee on Research Animal Care (SRAC), serving as the Institutional Animal Care and Use Committee (IACUC) for Massachusetts General Hospital (MGH), reviewed and approved all procedures outlined in this paper.

Micro PET/CT imaging was performed on anesthetized C57BL/6 mice using isoflurane to minimize discomfort during the procedure. Highly trained animal technicians closely monitored the safety and well‐being of the animals throughout the imaging process, while veterinary staff provided daily care. All mice were housed in social groups in cages designed to promote the physical and behavioral health of the individual animals. The mice had unlimited access to food and water, with additional nutritional supplements provided as recommended by the attending veterinary staff.

### Synthesis of 4‐(3‐bromopropoxy)‐5‐chloro‐2‐(4‐methoxyphenyl)‐6‐methylpyrimidine (2)

The synthesis of compound **2** followed the procedures we reported previously, with minor modifications.^[^ ^35^, ^43^ ^]^ To a mixture of 5‐chloro‐2‐(4‐methoxyphenyl)‐6‐methylpyrimidin‐4‐ol (compound **1**, 2.5 g, 10 mmol) and 1,3‐dibromopropane (4.0 g, 20 mmol) in acetone (25 mL), potassium carbonate (K_2_CO_3_, 2.76 g, 20 mmol) was added while stirring. The mixture was heated to reflux at 58 °C for 2.0 h, with progress monitored using thin‐layer chromatography (TLC). After cooling to room temperature, the mixture was filtered, and the solvent was evaporated under reduced pressure. The crude product was purified using flash chromatography (hexane/ethyl acetate = 20/1) to yield 4‐(3‐bromopropoxy)‐5‐chloro‐2‐(4‐methoxyphenyl)‐6‐methylpyrimidine (compound **2**) as a colorless oil (1.73 g, 46.5% yield). The ^1^H NMR (500 MHz, Chloroform‐*d*) data for compound **2** are as follows: δ 8.41–8.30 (m, 2H), 7.00–6.93 (m, 2H), 4.68 (t, *J* = 6.0 Hz, 2H), 3.87 (s, 3H), 3.62 (t, *J* = 6.5 Hz, 2H), 2.59 (s, 3H), 2.49–2.36 (m, 2H). The ^13^C NMR (126 MHz, Chloroform‐*d*) data for compound **2** are as follows: δ 164.51, 163.87, 161.95, 160.30, 129.96, 129.59, 113.85, 113.25, 64.90, 55.47, 31.97, 29.78, and 22.22. LC–MS data: calculated [M+H]^+^ for C_15_H_16_BrClN_2_O_2_: 372.7; found [M+H]^+^: 372.6.

### Synthesis of 5‐Chloro‐2‐(4‐Methoxyphenyl)‐4‐Methyl‐6‐(3‐(Piperidin‐1‐yl)Propoxy)Pyrimidine (CNY‐01)

The synthesis of standard **CNY‐01** was conducted according to previously reported procedures with minor modifications.^[^ ^35^, ^43^ ^]^ To a mixture of 4‐(3‐bromopropoxy)‐5‐chloro‐2‐(4‐methoxyphenyl)‐6‐methylpyrimidine (compound **2**, 0.74 g, 2 mmol) and piperidine (0.19 g, 2.2 mmol) in acetonitrile (10 mL), cesium carbonate (Cs_2_CO_3_, 1.3 g, 4 mmol) was added while stirring. The mixture was heated to reflux at 82 °C for 4.0 h, with progress monitored using thin‐layer chromatography (TLC). Once the reaction mixture cooled to room temperature, it was filtered and the solvent was evaporated under reduced pressure. The crude product was purified using flash chromatography (dichloromethane/methanol = 10/1) to yield 5‐chloro‐2‐(4‐methoxyphenyl)‐4‐methyl‐6‐(3‐(piperidin‐1‐yl)propoxy)pyrimidine (**CNY‐01**) as a white solid (0.66 g, 87.8% yield). The ^1^H NMR (500 MHz, Chloroform‐*d*) data for compound **3** are as follows: δ 8.38–8.30 (m, 2H), 7.00–6.92 (m, 2H), 4.58 (t, *J* = 6.5 Hz, 2H), 3.87 (s, 3H), 2.58 (s, 3H), 2.55–2.28 (m, 6H), 2.11–2.00 (m, 2H), 1.65–1.54 (m, 4H), 1.45 (d, *J* = 6.0 Hz, 2H). The ^13^C NMR (126 MHz, Chloroform‐*d*) data for compound 3 are as follows: δ 164.20, 164.15, 161.83, 160.25, 129.91, 129.80, 113.80, 113.32, 66.10, 56.03, 55.46, 54.75, 26.45, 26.09, 24.53, and 22.23. LC–MS data: calculated [M+H]^+^ for C_20_H_26_ClN_3_O_2_: 376.2; found [M+H]^+^: 376.2.

### Synthesis of 4‐(3‐Bromopropoxy)‐5‐Chloro‐2‐(4‐Chlorophenyl)‐6‐Methylpyrimidine (4)

The synthesis of compound 5 followed previously reported procedures with minor modifications.^[^ ^35^, ^43^ ^]^ To a mixture of 5‐chloro‐2‐(4‐chlorophenyl)‐6‐methylpyrimidin‐4‐ol (compound **3**, 2.55 g, 10 mmol) and 1,3‐dibromopropane (4.0 g, 20 mmol) in acetone (25 mL), potassium carbonate (K_2_CO_3_, 2.76 g, 20 mmol) was added while stirring. The mixture was heated to reflux at 58 °C for 2.0 h, with progress monitored using thin‐layer chromatography (TLC). After cooling to room temperature, the mixture was filtered and the solvent evaporated under reduced pressure. The crude product was purified using flash chromatography (hexane/ethyl acetate = 10/1) to yield 4‐(3‐bromopropoxy)‐5‐chloro‐2‐(4‐chlorophenyl)‐6‐methylpyrimidine (compound **4**) as a pale‐yellow oil (1.8 g, 47.8% yield). The ^1^H NMR (500 MHz, Chloroform‐*d*) data for compound **4** are as follows: δ 8.37–8.31 (m, 2H), 7.45–7.41 (m, 2H), 4.69 (t, *J* = 6.0 Hz, 2H), 3.63 (t, *J* = 6.4 Hz, 2H), 2.61 (s, 3H), and 2.47–2.38 (m, 2H). The ^13^C NMR (126 MHz, Chloroform‐*d*) data for compound **5** are as follows: δ 164.76, 164.07, 159.47, 137.01, 135.37, 129.64, 128.76, 114.40, 65.09, 31.88, 29.67, and 22.20. LC–MS data: calculated [M+H]^+^ for C_14_H_13_BrCl_2_N_2_O: 377.1; found [M+H]^+^: 377.0.

### Synthesis of tert‐Butyl 4‐(3‐((5‐Chloro‐2‐(4‐Chlorophenyl)‐6‐Methylpyrimidin‐4‐yl)Oxy)Propyl)piperazine‐1‐Carboxylate (5)

The synthesis of compound **5** was performed followed the procedures we reported previously with minor modifications.^[^ ^35^, ^43^ ^]^ To a mixture of 4‐(3‐bromopropoxy)‐5‐chloro‐2‐(4‐methoxyphenyl)‐6‐methylpyrimidine (compound **4**, 0.74 g, 2 mmol) and tert‐butyl piperazine‐1‐carboxylate (0.41 g, 2.2 mmol) in acetonitrile (10 mL), cesium carbonate (Cs_2_CO_3_, 1.3 g, 4 mmol) was added while stirring. The mixture was heated to reflux at 82 °C for 4.0 h, with progress monitored using thin‐layer chromatography (TLC). Once the reaction mixture cooled to room temperature, it was filtered, and the solvent evaporated under reduced pressure. The crude product was purified using flash chromatography (dichloromethane/methanol = 10/1) to yield tert‐butyl 4‐(3‐((5‐chloro‐2‐(4‐chlorophenyl)‐6‐methylpyrimidin‐4‐yl)oxy)propyl)piperazine‐1‐carboxylate (compound **5**) as a colorless oil (0.75 g, 77.9% yield). The ^1^H NMR (500 MHz, Chloroform‐*d*) data for compound **5** are as follows: δ 8.39–8.24 (m, 2H), 7.48–7.37 (m, 2H), 4.60 (t, *J* = 6.4 Hz, 2H), 3.44 (t, *J* = 5.0 Hz, 4H), 2.64–2.36 (m, 9H), 2.11–2.00 (m, 2H), and 1.46 (s, 9H). The ^13^C NMR (126 MHz, Chloroform‐*d*) data for compound **7** are as follows: δ 164.53, 164.29, 159.42, 154.83, 136.91, 135.53, 129.59, 128.72, 114.43, 65.86, 55.16, 53.13, 28.52, 26.21, and 22.21. LC–MS data: calculated [M+H]^+^ for C_23_H_30_Cl_2_N_4_O_3_: 482.4; found [M+H]^+^: 482.4.

### Synthesis of 5‐Chloro‐2‐(4‐Chlorophenyl)‐4‐Methyl‐6‐(3‐(4‐Methylpiperazin‐1‐yl)Propoxy)Pyrimidine (CNY‐02)

The synthesis of standard **CNY‐02** followed previously reported procedures with minor modifications.^[^ ^35^, ^43^ ^]^ To a mixture of 4‐(3‐bromopropoxy)‐5‐chloro‐2‐(4‐methoxyphenyl)‐6‐methylpyrimidine (compound **5**, 0.74 g, 2 mmol) and 1‐methylpiperazine (0.22 g, 2.2 mmol) in acetonitrile (10 mL), cesium carbonate (Cs_2_CO_3_, 1.3 g, 4 mmol) was added while stirring. The mixture was heated to reflux at 82 °C for 4.0 h, with progress monitored using thin‐layer chromatography (TLC). Once the reaction mixture cooled to room temperature, it was filtered, and the solvent evaporated under reduced pressure. The crude product was purified using flash chromatography (dichloromethane/methanol = 10/1) to yield 5‐chloro‐2‐(4‐chlorophenyl)‐4‐methyl‐6‐(3‐(4‐methylpiperazin‐1‐yl)propoxy)pyrimidine (**CNY‐02**) as a colorless oil (0.64 g, 80.5% yield). The ^1^H NMR (500 MHz, Chloroform‐*d*) data for **CNY‐02** are as follows: δ 8.37–8.30 (m, 2H), 7.45–7.40 (m, 2H), 4.60 (t, *J* = 6.4 Hz, 2H), 2.75–2.34 (m, 13H), 2.32 (s, 3H), and 2.11–2.00 (m, 2H). The ^13^C NMR (126 MHz, Chloroform‐*d*) data for compound **6** are as follows: δ 164.48, 164.32, 159.40, 136.87, 135.56, 129.60, 128.70, 114.46, 66.03, 55.24, 55.13, 53.33, 46.16, 26.34, and 22.21. LC–MS data: calculated [M+H]^+^ for C19H24Cl2N4O2: 396.3; found [M+H]^+^: 396.2.

### Synthesis of 4‐(5‐Chloro‐4‐Methyl‐6‐(3‐(Piperidin‐1‐yl)Propoxy)Pyrimidin‐2‐yl)Phenol (Precursor 1)

The synthesis of precursor 1 followed the procedures that reported previously, with minor modifications.^[^ ^43^ ^]^

Briefly, under a nitrogen (N_2_) atmosphere, a solution of compound **3** (0.375 g, 1.0 mmol) in dichloromethane (8.0 mL) was kept in an acetone‐dry ice bath at −78 °C. Next, 6 mL of a 1.0 mol L^−1^ boron tribromide solution in dichloromethane was carefully added to the stirring solution, which was maintained at −78 °C for 2.0 h. As the boron tribromide solution was added, a pale‐yellow precipitate formed. The reaction mixture was then gradually warmed to room temperature and stirred overnight. The mixture was carefully hydrolyzed by shaking with 40 mL of water, precipitating a white solid. This solid was dissolved by the addition of 30 mL of dichloromethane. The organic layer was separated and extracted with 20 mL of 2.0 mol L^−1^ sodium hydroxide. The alkaline extract was neutralized with dilute hydrochloric acid and extracted with dichloromethane (3 × 10 mL). The combined organic layers were dried with anhydrous magnesium sulfate. The filtrate was then evaporated under reduced pressure, and the crude product was purified using flash chromatography (dichloromethane/methanol = 10/1) to yield 4‐(5‐chloro‐4‐methyl‐6‐(3‐(piperidin‐1‐yl)propoxy)pyrimidin‐2‐yl)phenol (precursor 1) as a white solid (0.13 g, 36.5%). The ^1^H NMR (500 MHz, DMSO‐*d*_6_) data for precursor **1** are as follows: δ 9.98 (s, weak signal for the phenol group), 8.19–8.10 (m, 2H), 6.86–6.74 (m, 2H), 4.50 (t, *J* = 6.6 Hz, 2H), 2.46 (s, 3H, overlap with DMSO‐d6 signal), 2.40–2.19 (m, 6H), 1.87 (p, *J* = 6.8 Hz, 2H), 1.43 (p, *J* = 5.5 Hz, 4H), and 1.38–1.24 (m, 2H). The ^13^C NMR (126 MHz, DMSO‐*d*_6_) data for precursor **1** are as follows: δ 164.38, 164.06, 160.78, 160.25, 130.25, 127.62, 115.87, 112.41, 66.22, 55.55, 54.61, 26.26, 26.12, 24.66, and 22.43. LC–MS data: calculated [M+H]^+^ for C_19_H_24_ClN_3_O_2_: 362.2; found [M+H]^+^: 362.2.

### Synthesis of 5‐Chloro‐2‐(4‐Chlorophenyl)‐4‐Methyl‐6‐(3‐(Piperazin‐1‐yl)Propoxy)Pyrimidine (Precursor 2)

The synthesis of precursor **2** followed previously reported procedures with minor modifications.^[^ ^43^ ^]^ In brief, compound **5** (0.481 g, 1 mmol) was dissolved in a mixture of trifluoroacetic acid (TFA, 3.0 mL) and dichloromethane (6.0 mL). The reaction mixture was stirred at room temperature for 2.0 h, and then the solvent was evaporated under reduced pressure. The residue was dissolved in dichloromethane and neutralized with tripropylamine. The crude product mixture was then purified using flash chromatography (dichloromethane/methanol = 10/1) to yield 5‐chloro‐2‐(4‐chlorophenyl)‐4‐methyl‐6‐(3‐(piperazin‐1‐yl)propoxy)pyrimidine (precursor **2**) as a white solid (0.32 g, 83.9% yield). The ^1^H NMR (500 MHz, Chloroform‐*d*) data for precursor **2** are as follows: δ 8.39–8.29 (m, 2H), 7.46–7.37 (m, 2H), 4.59 (t, *J* = 6.3 Hz, 2H), 3.09 (t, *J* = 5.0 Hz, 4H), and 2.80–2.36 (m, 10H), 2.09–1.99 (m, 2H). The ^13^C NMR (126 MHz, Chloroform‐*d*) data for precursor **2** are as follows: δ 164.59, 164.24, 159.44, 136.94, 135.50, 129.59, 128.74, 114.40, 65.62, 55.08, 51.66, 44.61, 26.05, and 22.21. LC–MS data: calculated [M+H]^+^ for C_18_H_22_Cl_2_N_4_O: 381.1; found [M+H]^+^: 381.2.

### Ex Vivo Binding Assays for *σ*Rs

The following specific radioligands and tissue sources were used: (a) *σ*_1_R, [^3^H] (+)‐pentazocine, Dunkin Hartley guinea pig brain membranes; (b) *σ*_2_R, [^3^H]‐DTG, Dunkin Hartley guinea pig brain membranes. Membrane preparation of brain tissue was performed following the previously reported method.4 Generally, for *σ*_1_R binding test, guinea pig brain membranes protein (5‐10 mg), [^3^H]‐(+)‐pentazocine (4 nm), test compounds solution in various concentrations (10^−5^ to 10^−10^ m) were incubated at 25 °C for 180 min in Tris‐HCl buffer (pH 8.0). For *σ*_2_R binding study, the membrane suspension was incubated with [^3^H]‐DTG (3 nm), different concentration test compounds, within 400 nm (+)‐SKF10047 to block *σ*_1_R. Nonspecific binding was conducted in both binding assays under similar conditions, adding 10 mm haloperidol. After incubation, the mixture was rapidly filtrated through Whatman GF/B glass filters, washed twice and transferred to scintillation vials, and the radioactivity bound was measured by a Beckman LS 6500 liquid scintillation counter. Inhibition constants (K_i_) were calculated based on the Cheng and Prusoff equation after the IC_50_ values were obtained.

### Radiosynthesis of [^11^C]CNY‐01

The radiosynthesis was performed according to previously reported procedures, with minor modifications.^[^ ^43^, ^51^, ^52^ ^]^ [^11^C]CH_3_I was directly trapped in a TRACERlab FX‐M synthesizer reactor (General Electric) preloaded with precursor **1** (0.6 mg) and NaOH (8.0 mg) in DMF (0.3 mL). The mixture was heated at 100 °C for 3 min, followed by the addition of a solution of 0.1% TFA in water (1.2 mL). The reaction mixture was then purified using reverse‐phase semipreparative HPLC (Agilent Eclipse XDB‐C18, 5 µm, 250 mm × 9.4 mm, flow rate = 5.0 mL min^−1^, mobile phase = 0.1% TFA in water/0.1% TFA in acetonitrile, 60/40, v/v), and the desired fraction was collected. The final product was reformulated by loading it onto a solid‐phase exchange (SPE) C‐18 cartridge, rinsing with water (5.0 mL), eluting with ethanol (1.0 mL), and diluting with saline solution (0.9%, 9.0 mL). The average synthesis time from the end of cyclotron bombardment to the end of synthesis (EOS) was ≈40–50 min. The average radiochemical yield of [^11^C]CNY‐01 was 8–15% (nondecay corrected to trapped [^11^C]CH_3_I). Chemical and radiochemical purities were ≥95% with a specific activity of 59.2 ± 7.4 GBq µmol^−1^ (EOB).

### Radiosynthesis of [^11^C]CNY‐02

The preparation method for [^11^C]CNY‐02 was similar to that of [^11^C]CNY‐01, with minor modifications.^[^ ^43^, ^51^, ^52^ ^]^ [^11^C]CH_3_I was directly trapped in a TRACERlab FX‐M synthesizer reactor (General Electric) preloaded with precursor **2** (0.6 mg) and K_2_CO_3_ (8.0 mg) in anhydrous DMF (0.3 mL). The mixture was heated to 120 °C for 3 min, followed by the addition of a solution of 0.1% TFA in water (1.2 mL). The reaction mixture was then purified using reverse‐phase semipreparative HPLC (Agilent Eclipse XDB‐C18, 5 µm, 250 mm × 9.4 mm, flow rate = 5.0 mL min^−1^, mobile phase = 0.1% TFA in water/0.1% TFA in acetonitrile, 66/34, v/v), and the desired fraction was collected. The final product was reformulated by loading it onto a solid‐phase exchange (SPE) C‐18 cartridge, rinsing with water (5.0 mL), eluting with ethanol (1.0 mL), and diluting with saline solution (0.9%, 9.0 mL). The preparation of [^11^C]CNY‐02, including formulation, was completed within 50 to 60 min after the end of bombardment (EOB). The average radiochemical yield was 10–15% (nondecay corrected to trapped [^11^C]CH_3_I, EOS). The chemical and radiochemical purities were ≥95%, with a specific activity of 45.1 ± 3.2 GBq µmol^−1^ (EOB).

### Mice PET/CT Acquisition and Post‐Processing

The PET/CT studies in mice followed previously reported procedures with minor modifications.^[^ ^53^, ^54^ ^]^ Mice (wild‐type: C57BL/6, male, 6‐month‐old; AD transgenic: 5xFAD, male, 6‐month‐old, n = 4 for each group) were anesthetized with inhalational isoflurane (Patterson Vet Supply, Inc., Greeley, CO, USA) at 2.0% in a carrier of 2 L min^−1^ medical oxygen, and maintained at 1.0% isoflurane during the imaging scan. Mice were injected with radiotracer (200 µL) via lateral tail vein catheterization at the start of the dynamic PET scan (Gamma Medica, Northridge, CA, USA). For blocking studies, the blocking agent was administered via the tail vein 5 min before radiotracer injection. Dynamic PET acquisition lasted 60 min and was followed by computed tomography (CT) for anatomic co‐registration. PET data were reconstructed using a 3D‐MLEM method, resulting in a full width at half‐maximum resolution of 1 mm. Reconstructed images were exported from the scanner in DICOM format and were analyzed using PMOD (PMOD 4.01).

### NHP PET/MR Imaging

The PET/MR imaging protocol in non‐human primates (NHP) followed our previous work.^[^ ^55^, ^56^ ^]^ A male rhesus macaque (8.55 kg) was anesthetized with an intramuscular injection of xylazine (0.5–2.0 mg kg^−1^) and ketamine (10 mg kg^−1^), followed by endotracheal intubation. V‐ and A‐lines were inserted, and anesthesia was maintained using isoflurane. [^11^C] CNY‐01 was administered after antecubital catheterization, with an arterial line prepared for radiometabolite analysis. In the self‐blocking scan, the same animal (6.80 kg) was scanned with a 10‐min pre‐treatment of 0.5 mg kg^−1^ unlabeled CNY‐01. PET/MR imaging data were acquired using a Siemens TIM‐Trio scanner (3T) equipped with a Siemens BrainPET insert module and an in‐house‐built 8‐channel head coil. The dynamic scan began, followed by administration of [^11^C] CNY‐01 (5.1 mCi). The list mode data were reconstructed using the 3D ordinary Poisson expectation‐maximization algorithm (16 subsets and 6 iterations) with detector efficiency, decay, dead time, random coincidences, scatter, and attenuation corrections with gradually increasing time frams. All images were reconstructed into 153 slices with 256 × 256 pixels and 1.25‐mm isotropic voxel size. High‐resolution structural MRI scans were acquired by using a multi‐echo magnetization‐prepared rapid gradient echo (ME‐MPRAGE) sequence with parameters as follows: TR = 2400 ms; TE1/TE2/TE3/TE4 = 1.57/3.29/5.01/6.73 ms; inversion time = 1200 ms; flip angle = 9°; spatial resolution = 1 mm isotropic.

For macaque brain PET/MR imaging analysis, we applied the INIA19 Template^[^ ^57^ ^]^ and NeuroMaps Atlas in accordance with our prior work.^[^ ^58^ ^]^ In brief, reconstructed PET images were registered to the INIA19 Primate Brain Atlas^[^ ^57^ ^]^ with the following processing steps^[^ ^59^ ^]^: The high‐resolution T1‐weighted MR image, co‐registered with the PET image, was first affine‐registered to the INIA19 atlas. By using the same transformation, dynamic PET data was registered to the atlas. A spatial smoothing with a 6‐mm FWHM Gaussian kernel was applied to PET images. Twenty ROIs determined in the INIA19 atlas were used to derive regional TACs. These ROIs include caudate, putamen, nucleus accumbens, ventral tegmental area, thalamus, hypothalamus, amygdala, hippocampus, insula, anterior cingulate cortex, posterior cingulate cortex, occipital gyrus, midbrain, sensory cortex, motor cortex, inferior frontal cortex, orbitofrontal cortex, cerebellum, white matter, and whole brain. To test which model and approach best fits [^11^C]CNY‐01 kinetic, we performed kinetic analysis using regional TACs with the one‐ and two‐tissue compartmental model (1TCM and 2TCM) or Logan graphical analysis (LGA; assuming T* = 40 min)^[^ ^59^ ^]^ implemented in PMOD 3.9 (PMOD Technologies Ltd., Zurich, Switzerland) with the metabolite‐corrected arterial plasma as an input function, fitting the blood volume fraction and a delay term for the plasma input function to calculate the V_T_ for all ROIs.

### NHP Plasma and Metabolite Analysis

During the PET/MR scanning, macaque arterial blood samples were collected synchronously. Metabolite extraction and analysis were based on published methods.^[^ ^54^, ^60^ ^]^ Briefly, the blood samples were centrifuged to isolate plasma. Next, protein precipitation was achieved by mixing the plasma with acetonitrile (v/v = 1). The mixture was then centrifuged again to obtain protein‐free plasma. A 1 mL aliquot of the protein‐free supernatant was diluted in 4 mL of deionized water, and the radiometabolites were analyzed by HPLC. HPLC condition: Mobile Phase A: water + 0.1% formic acid; Mobile Phase B: acetonitrile + 0.1% formic acid; separation method = 95/5 – 50/50 A/B from 3–8 min linear gradient; 50/50–5/95 A/B from 8–10 min linear gradient; 5/95 A/B from 10–11 min isocratic; flow rate 2 mL min^−1^.

### Immunohistochemistry and Confocal Microscopy

The IHC analysis was performed following our previously reported methods.^[^ ^55^, ^61^, ^62^ ^]^ Brains from 5‐month‐old male WT and 5xFAD mice (n = 4) were sectioned into 40 µm thickness slices by microtome and carefully selected for immunostaining. First, brain sections were washed in PBS 4 times for 7 min each and then incubated in blocking solution (5% donkey serum + 0.3% Triton X‐100 in PBS) for 1 h at room temperature with gentle shaking to eliminate nonspecific staining. Next, sections were transferred into primary antibody solution, which contained 1:500 anti‐rat CD68 (MA5‐16674, Invitrogen), 1:200 anti‐rabbit *σ*_1_R (61 994, Cell Signaling Technology), and 1:600 anti‐mouse 6E10 (803 001, BioLegend) in 2.5% donkey serum + 0.3% Triton X‐100 in PBS and incubated overnight at 4 °C with gentle shaking. Thereafter, sections were washed in PBS 4 times for 7 min each, and then incubated in secondary antibody mix, which contained 1:500 anti‐rat Alexa Fluor 488 (712‐545‐153, Jackson Laboratories), 1:500 anti‐rabbit Alexa Fluor 594 (711‐585‐152, Jackson Laboratories), and 1:500 anti‐mouse Alexa Fluor 647 (A‐31571, Invitrogen) in blocking solution for 2 h at room temperature with gentle shaking and protected from light. Subsequently, sections were washed in PBS 3 times for 7 min each, carefully mounted on a glass slide with VECTASHIELD antifade mounting medium with DAPI and covered with a coverslip. Sections were imaged using a Nikon C2 confocal microscope at 4X objective magnifications and 40X objective magnifications in the cortex and dentate gyrus region of the hippocampus. Additionally, at 40X objective magnifications, Z‐stacks were acquired by capturing 1 image every 1 µm in a 40 µm section and then compiled and arranged into XY‐plane composite Movie S3 (Supporting Information), XYZ‐plane orthogonal projections, and 360° rotation Movie S4 (Supporting Information) using ImageJ processing software.

### Statistical Analysis

Confocal microscopic images were processed and analyzed using Image J software. All the results for different experiments in this work were shown as mean ± SEM. Statistical significances of different results were performed using two tailed Student's t‐test in GraphPad PRISM. Values were considered as significant when it comes *p* < 0.05.

## Conflict of Interest

The authors declare no conflict of interest.

## Supporting information



Supporting Information

Supplemental Movie 1

Supplemental Movie 2

Supplemental Movie 3

Supplemental Movie 4

## Data Availability

The data that support the findings of this study are available from the corresponding author upon reasonable request.
