# Supplementary material for: Development of Carbon‐11 Labeled Pyrimidine Derivatives as Novel Positron Emission Tomography (PET) Agents Enabling Brain Sigma‐1 Receptor Imaging
Source: Adv Sci (Weinh). 2025 Apr 17;12(21):2414827. doi: 10.1002/advs.202414827 (PMC12140300; doi:10.1002/advs.202414827)

**Supporting Information**

**Development of carbon-11 labeled pyrimidine derivatives as novel positron emission tomography (PET) agents enabling brain sigma-1 receptor imaging**

Ping Bai^1,2,3,4,5*^, Ashley Gomm^6^, Chi-Hyeon Yoo^7^, Prasenjit Mondal^6^, Fleur Marie Lobo^6^, Hui Meng^1,2,3,4,5^, Yanting Zhou^1,2,3,4,5^, Weiyao Xie^1,2,3,4,5^, Hsiao-Ying Wey^7^, Rudolph E. Tanzi^6^, Can Zhang^6*^, Changning Wang^7*^, Yu Lan^8*^

^1^ Department of Respiratory and Critical Care Medicine, Targeted Tracer Research and Development Laboratory, West China Hospital, Sichuan University, Chengdu, Sichuan 610041, China

^2^ Institute of Respiratory Health, Targeted Tracer Research and Development Laboratory, Frontiers Science Center for Disease-related Molecular Network, West China Hospital, Sichuan University, Chengdu, Sichuan 610041, China

^3^ Precision Medicine Center, Precision Medicine Key Laboratory of Sichuan Province, West China Hospital, Sichuan University, Chengdu, Sichuan 610041, China

^4^ The Research Units of West China, Chinese Academy of Medical Sciences, West China Hospital, Chengdu, Sichuan 610041, China

^5^ State Key Laboratory of Respiratory Health and Multimorbidity, West China Hospital, Chengdu, Sichuan 610041, China

^6^ Genetics and Aging Research Unit, McCance Center for Brain Health, MassGeneral Institute for Neurodegenerative Disease, Department of Neurology, Massachusetts General Hospital, Harvard Medical School, 114 16th Street, Charlestown, MA 02129, United States

^7^ Athinoula A. Martinos Center for Biomedical Imaging, Department of Radiology, Massachusetts General Hospital, Harvard Medical School, Charlestown, MA 02129, United States

^8^ Department of Pharmacy, Renmin Hospital of Wuhan University, Wuhan, Hubei 430060, China

**Contents**

Table S1. Off-target profile of CNY-01 and CNY-02 using radioligand assays……………………………… S3

Table S2. Regional volume of distribution (V_T_) values of [^11^C]CNY-01 obtained from a male NHP brain following 10-min pretreatment of unlabeled CNY-01. ………………………………………………………………… S6

Figure S1. Fluorescent immunohistochemistry analysis of *σ*_1_R in WT nontransgenic and 5xFAD transgenic animal brain sections…………………………………………………………………………………………………… S7

Figure S2. Fluorescent immunohistochemistry analysis of *σ*_1_R in WT nontransgenic and 5xFAD transgenic animal brain sections…………………………………………………………………………………………………… S8

Figure S3. XY plane composite movies of fluorescent immunohistochemistry analysis for *σ*_1_R in WT and 5xFAD transgenic animal brain sections………………………………………………………………………………… S9

Figure S4. 360 rotation movies of fluorescent immunohistochemistry analysis for *σ*_1_R in WT and 5xFAD transgenic animal brain sections …………………………………………………………………………………… S10

Figure S5. PET/MR imaging studies of [^11^C]CNY-01 in NHP with a 10-min pretreatment of unlabeled CNY-01 …………………………………………………………………………………………………………………………………… S11

NMR spectra for the synthesized compounds……………………………………………………………………………… S12

**Table S1**. Off-target profile of CNY-01and CNY-02 using radioligand assays^a^.

| Receptor | Compound | Mean %（inhibition） | Ki (nM) |
| --- | --- | --- | --- |
| 5-HT1A | CNY-01 | 17.46 |  |
|  | CNY-02 | -2.49 |  |
| 5-HT1B | CNY-01 | 79.76 | 3202.76 |
|  | CNY-02 | 60.3 | 3744.51 |
| 5-HT1D | CNY-01 | 91.93 | 2670.31 |
|  | CNY-02 | 54.55 | 7279.50 |
| 5-HT1E | CNY-01 | 31.25 |  |
|  | CNY-02 | 27.78 |  |
| 5-HT2A | CNY-01 | 36.28 |  |
|  | CNY-02 | 85.5 | 465.59 |
| 5-HT2B | CNY-01 | 77.12 | 914.08 |
|  | CNY-02 | 90.38 | 529.89 |
| 5-HT2C | CNY-01 | -5.06 |  |
|  | CNY-02 | 89.38 | 268.39 |
| 5-HT3 | CNY-01 | 7.61 |  |
|  | CNY-02 | -1.23 |  |
| 5-HT5A | CNY-01 | 31.93 |  |
|  | CNY-02 | 29.43 |  |
| 5-HT6 | CNY-01 | 30.05 |  |
|  | CNY-02 | 28.1 |  |
| 5-HT7A | CNY-01 | 59.27 | 4703.74 |
|  | CNY-02 | -4.02 |  |
| Alpha1A | CNY-01 | 21.62 |  |
|  | CNY-02 | -1.08 |  |
| Alpha1B | CNY-01 | -11.38 |  |
|  | CNY-02 | -26.12 |  |
| Alpha1D | CNY-01 | 22.98 |  |
|  | CNY-02 | -14.82 |  |
| Alpha2A | CNY-01 | 55.94 | 2373.85 |
|  | CNY-02 | 47.62 |  |
| Alpha2B | CNY-01 | 93.11 | 1350.85 |
|  | CNY-02 | 68.42 | 3390 |
| Alpha2C | CNY-01 | 85.11 | 430.37 |
|  | CNY-02 | 52.42 | 3767.04 |
| Beta1 | CNY-01 | 7.15 |  |
|  | CNY-02 | 3.55 |  |
| Beta2 | CNY-01 | 4.03 |  |
|  | CNY-02 | 11.68 |  |
| Beta3 | CNY-01 | 9.11 |  |
|  | CNY-02 | -3.01 |  |
| BZP Rat Brain Site | CNY-01 | 10.99 |  |
|  | CNY-02 | 23.26 |  |
| D1 | CNY-01 | 19.02 |  |
|  | CNY-02 | 52.14 | 2341.54 |
| D2 | CNY-01 | 4.43 |  |
|  | CNY-02 | -0.57 |  |
| D3 | CNY-01 | 65.78 | 521.39 |
|  | CNY-02 | 66.07 | 4913.88 |
| D4 | CNY-01 | 48.03 |  |
|  | CNY-02 | 17.82 |  |
| D5 | CNY-01 | 27.19 |  |
|  | CNY-02 | 57.98 | > 10000 |
| DAT | CNY-01 | 20.92 |  |
|  | CNY-02 | 33.78 |  |
| DOR | CNY-01 | 3.77 |  |
|  | CNY-02 | 1.3 |  |
| GABAA | CNY-01 | 25.49 |  |
|  | CNY-02 | 32.06 |  |
| H1 | CNY-01 | 93.15 | 667.05 |
|  | CNY-02 | 43.67 |  |
| H2 | CNY-01 | 89.66 | 709.18 |
|  | CNY-02 | 84.87 | 1236.60 |
| H3 | CNY-01 | 62.75 | 1062.87 |
|  | CNY-02 | 42.85 |  |
| H4 | CNY-01 | 24 |  |
|  | CNY-02 | 23.26 |  |
| KOR | CNY-01 | 61.09 | 1060.53 |
|  | CNY-02 | 20.43 |  |
| M1 | CNY-01 | 22.33 |  |
|  | CNY-02 | 35.95 |  |
| M2 | CNY-01 | 36.43 |  |
|  | CNY-02 | 29.06 |  |
| M3 | CNY-01 | 16.34 |  |
|  | CNY-02 | 53.97 | 727.27 |
| M4 | CNY-01 | 20.61 |  |
|  | CNY-02 | 31.1 |  |
| M5 | CNY-01 | 59.06 | > 10000 |
|  | CNY-02 | 83.27 | 2502.61 |
| MOR | CNY-01 | -16.42 |  |
|  | CNY-02 | -14.39 |  |
| NET | CNY-01 | 50.35 | 2104.28 |
|  | CNY-02 | 0.14 |  |
| PBR | CNY-01 | 25.78 |  |
|  | CNY-02 | 26.03 |  |
| SERT | CNY-01 | 21.79 |  |
|  | CNY-02 | 42.97 |  |

^a^ For targets with inhibition rates greater than 50%, we tested the ligands' Ki values. Details of individual binding assay conditions and protocols are available at the NIMH PDSP website: https://pdsp.unc.edu/pdspweb/.

**Table S2.** Regional volume of distribution (V_T_) values of [^11^C]CNY-01 obtained from a male NHP brain following 10-min pretreatment of unlabeled CNY-01.

| **Region** | **Two tissue compartment model**  **(2TCM)** | **Logan graphical method**  **(T* = 40min)** |
| --- | --- | --- |
|  | V_T_ (ml/cm^3^) | V_T_ (ml/cm^3^) |
| Caudate | 24.46 | 28.10 |
| Putamen | 27.99 | 33.81 |
| Globus-pallidus | 26.67 | 33.31 |
| NAc | 26.92 | 33.22 |
| VTA | 19.47 | 26.74 |
| Thalamus | 25.02 | 30.88 |
| Hypothalamus | 18.97 | 24.54 |
| ACC | 25.84 | 29.37 |
| PCC | 23.16 | 27.31 |
| Insula | 26.77 | 31.86 |
| Midbrain | 25.70 | 34.11 |
| Sensory | 16.21 | 19.24 |
| Motor | 16.66 | 21.53 |
| Ifc | 18.09 | 20.73 |
| Ofc | 16.68 | 20.65 |
| Occg | 11.84 | 12.96 |
| Hippocampus | 18.46 | 24.15 |
| Amygdala | 29.18 | 34.47 |
| CBL | 18.25 | 21.96 |
| WB | 16.75 | 20.75 |
| WM | 21.29 | 25.98 |


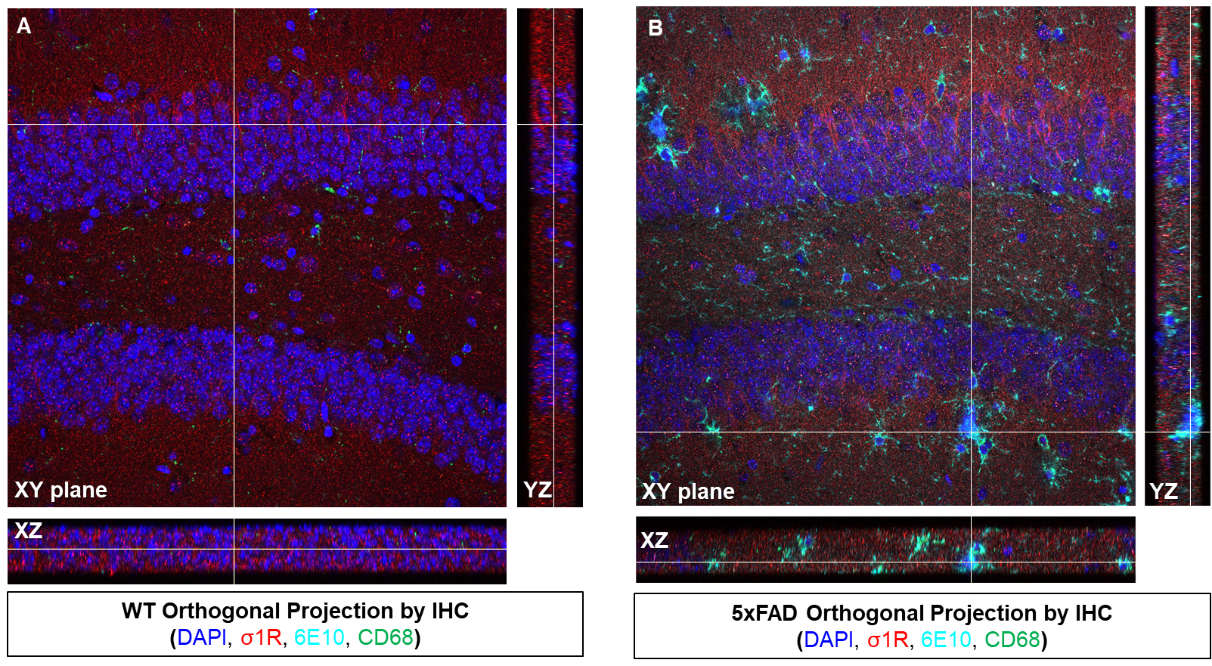


**Figure S1**. Fluorescent immunohistochemistry analysis of *σ*_1_R in WT nontransgenic and 5xFAD transgenic animal brain sections. Orthogonal projections captured by C2 microscope z-stack image at 40X magnification of hippocampus dentate gyrus (DG) region of 5-month-old male WT (A) and 5xFAD mice brain sections (B) stained with *σ*_1_R, in combination with DAPI (for nuclei), 6E10 (for amyloid deposition), and CD68 (microglia activation). The lines depicted the region of interest throughout the XY, XZ, and YZ planes, respectively showing perinucleus features of *σ*_1_R in WT mice and amyloid pathology-related absence of *σ*_1_R fluorescence signal and microglial activation in 5xFAD mice.

**
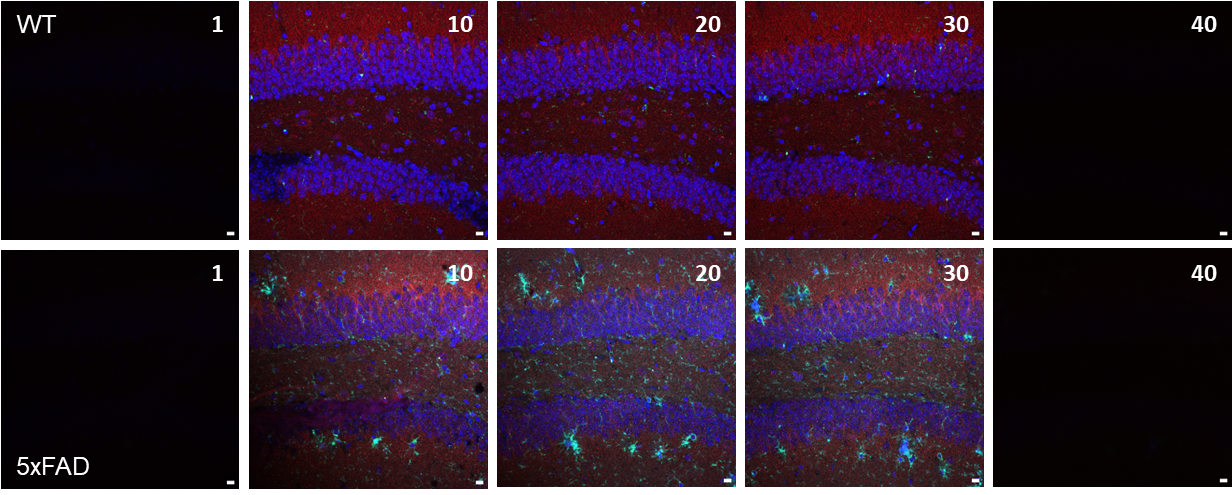
**

**Figure S2**. Fluorescent immunohistochemistry analysis of *σ*_1_R in WT nontransgenic and 5xFAD transgenic animal brain sections. Frame-by-frame montage captured by C2 microscope z-stack image at 40X magnification of hippocampus dentate gyrus (DG) region of 5-month-old male WT and 5xFAD mice brain sections stained with *σ*_1_R, in combination with DAPI (for nuclei), 6E10 (for amyloid deposition), and CD68 (microglia activation). Montage depicts merged fluorescence signals every 10 μm in 40 μm total brain tissue sections.


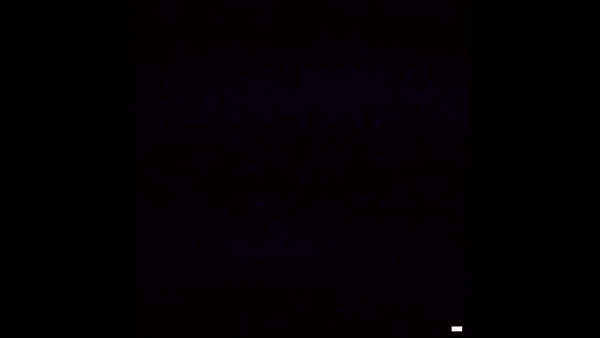


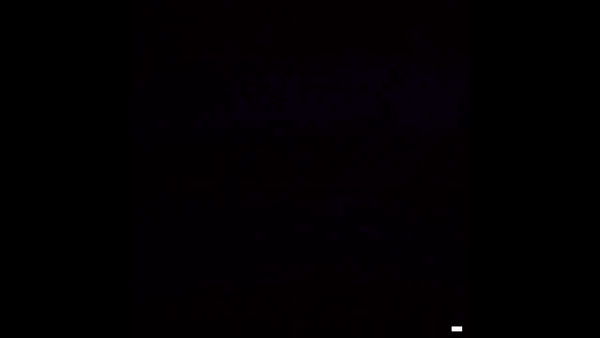


**Figure S3**. XY plane composite movies of fluorescent immunohistochemistry analysis for *σ*_1_R in WT (up) and 5xFAD transgenic (down) animal brain sections. Movies were captured by C2 microscope z-stack image at 40X magnification of hippocampus dentate gyrus region of 5-month-old male WT and 5xFAD mice brain sections stained with *σ*_1_R, in combination with DAPI (for nuclei), 6E10 (for amyloid deposition), and CD68 (microglia activation).

**
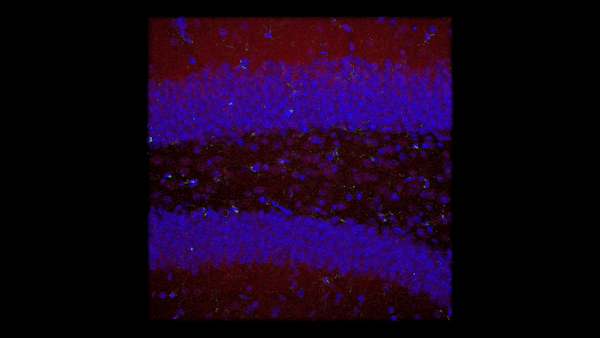

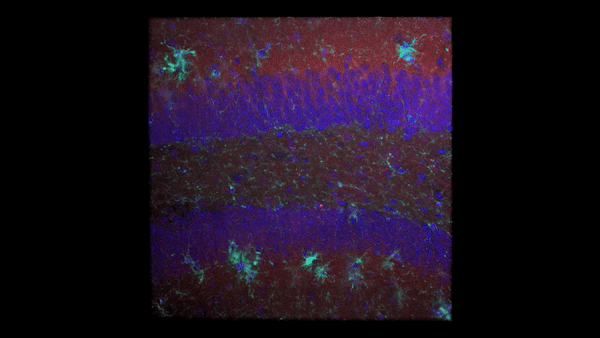
**

**Figure S4.** 360 rotation movies of fluorescent immunohistochemistry analysis for *σ*_1_R in WT (up) and 5xFAD transgenic animal (down) brain sections. Movies contained images in a volume captured by C2 microscope z-stack image at 40X magnification of hippocampus dentate gyrus region of 5-month-old male WT and 5xFAD mice brain sections stained with *σ*_1_R, in combination with DAPI (for nuclei), 6E10 (for amyloid deposition), and CD68 (microglia activation).


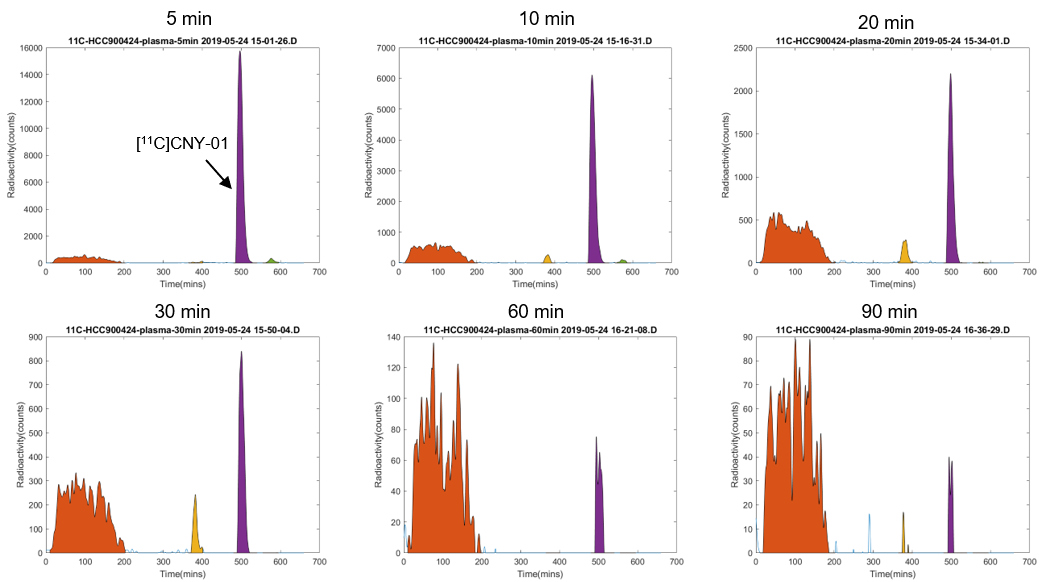


**Figure S5**. The HPLC chromatograms of metabolites in monkey plasma at time points 5, 10, 20, 30, 60, and 90 minutes.

**Figure S6.** PET/MR imaging studies of [^11^C]CNY-01 in NHP with a 10-min pretreatment of unlabeled CNY-01. (A) The parametric volume of interest (V_T_) map of [^11^C]CNY-01 was estimated by using the Logan graphical method with metabolite-corrected arterial input function illustrated with (B) parent fraction of [^11^C]CNY-01 in the blocking scan. (C) Time-activity curves of [^11^C]CNY-01 in brain regions of interest, illustrated with (D) radio metabolite-corrected plasm radioactivity.

# **NMR spectra for the synthesized compounds**.

**
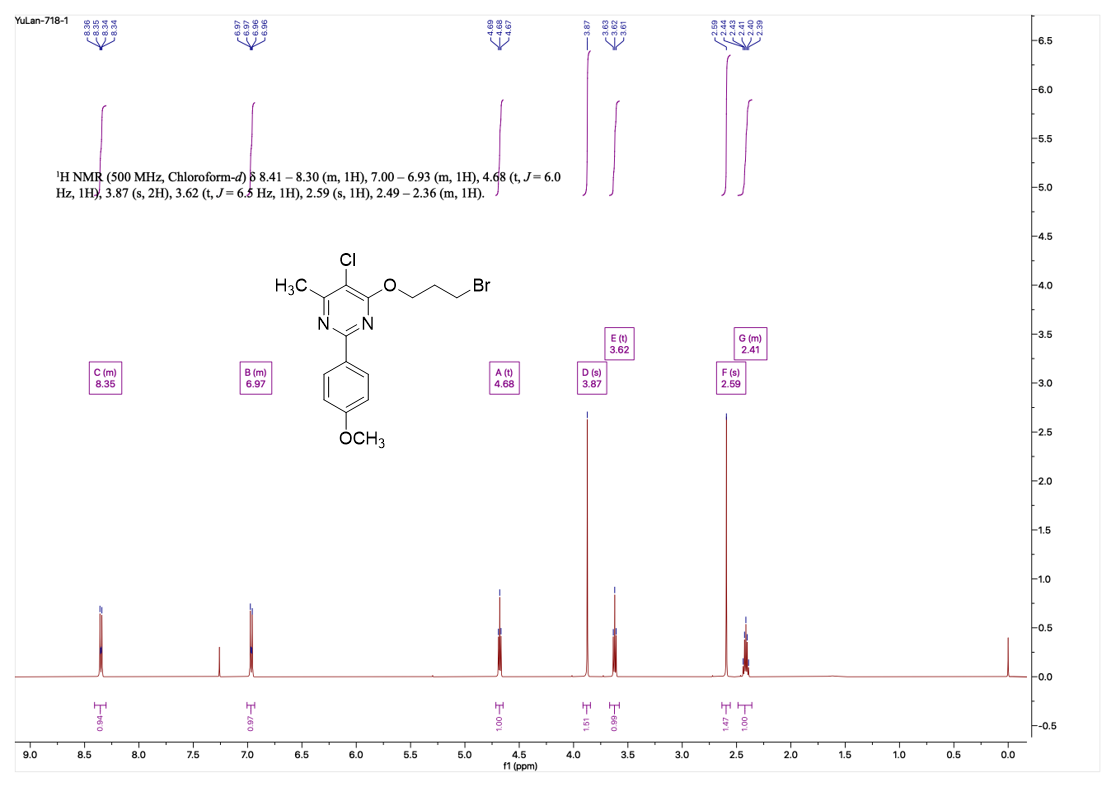
**

**
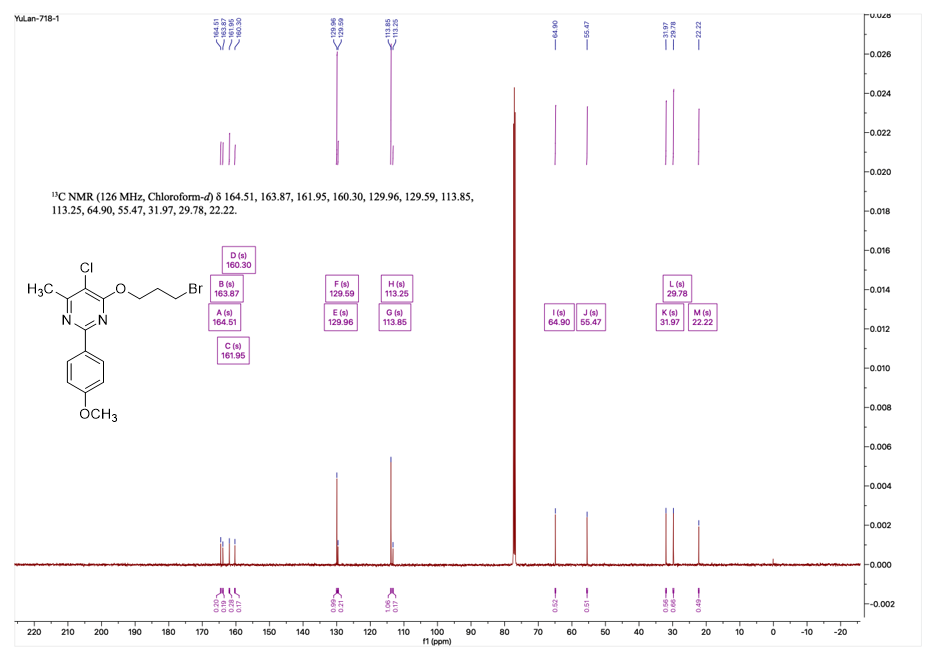
**


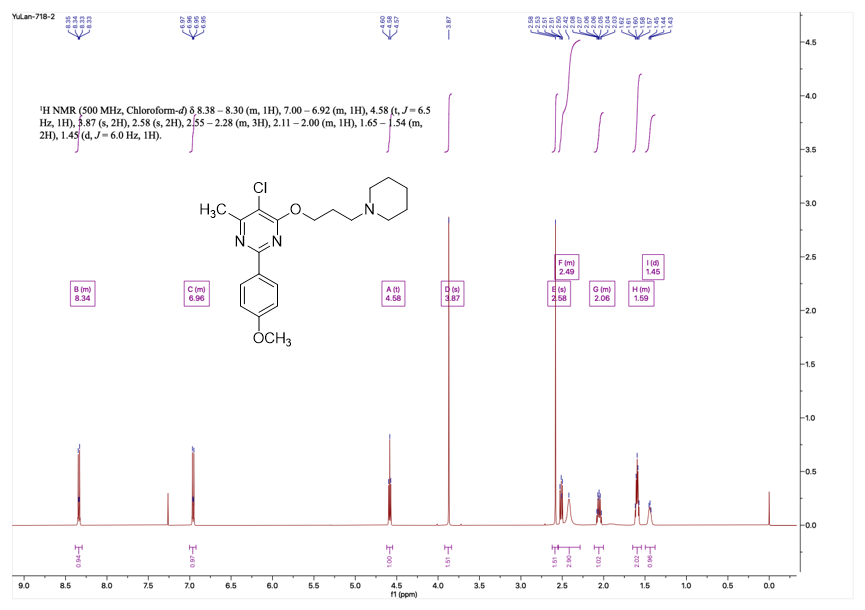


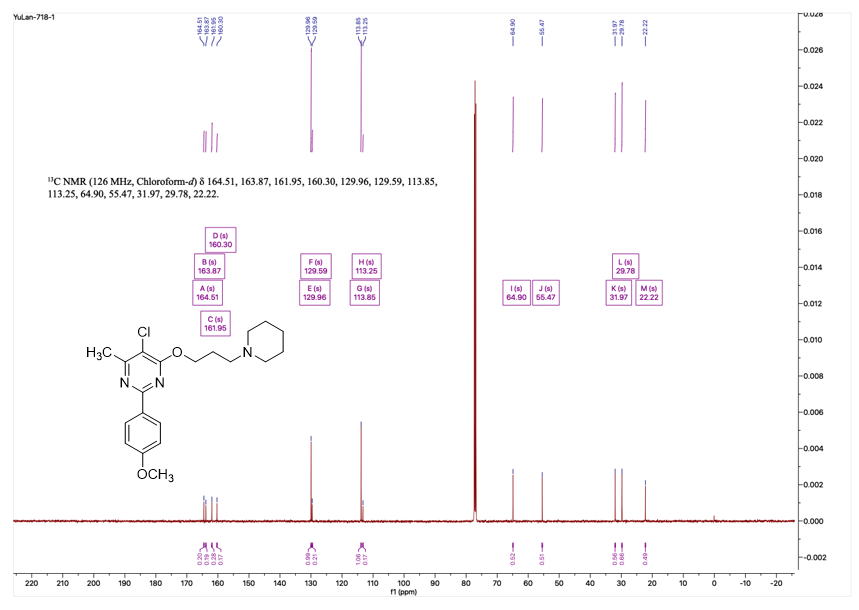


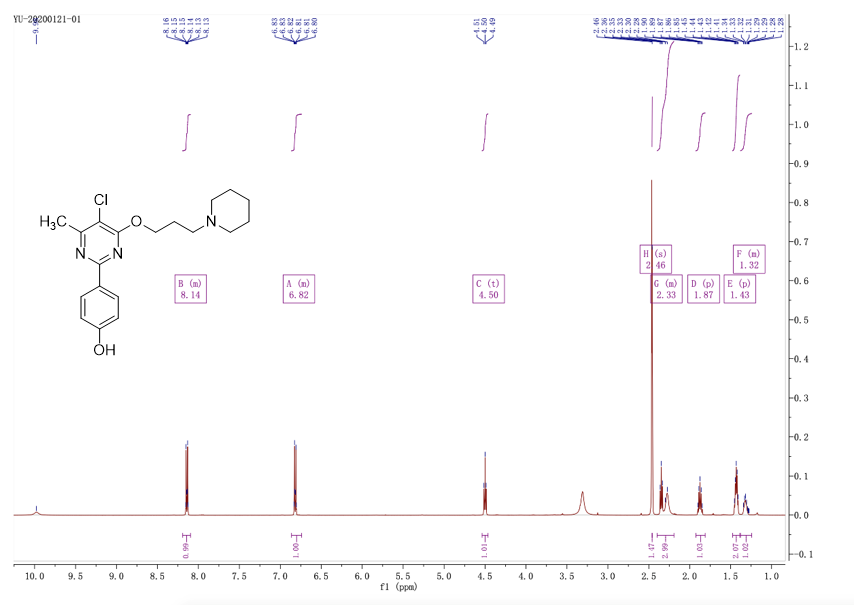


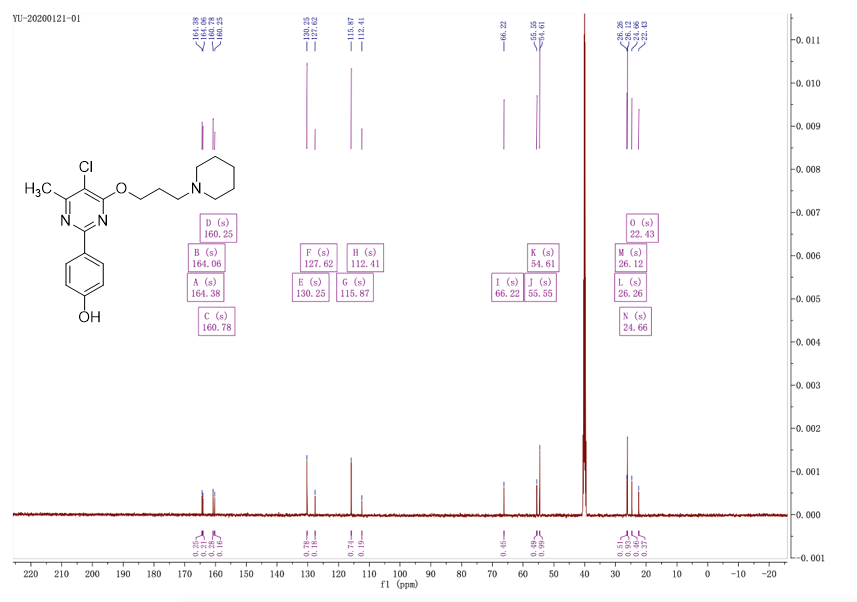

Supplement: Supplementary file 1 — Supporting Information [file ADVS-12-2414827-s004.docx]
